# Inflammatory cell death in cancer: linking pyroptosis, necroptosis, ferroptosis, PANoptosis, and antitumor immunity

**DOI:** 10.3389/fimmu.2026.2004224

**Published:** 2026-10-26

**Authors:** Shanshan Li, Zihan Zhou, Peng Li, Tao Zheng

**Affiliations:** 1 Department of Anesthesiology, The Third Affiliated Hospital of Kunming Medical University, Yunnan Cancer Hospital, Peking University Cancer Hospital Yunnan, Kunming, China; 2 Department of Clinical Pharmacy, Chongqing Medical University, Chongqing, China; 3 Department of Medical Affairs, Shandong Academy of Occupational Health and Occupational Medicine, Occupational Disease Hospital of Shandong First Medical University, Shandong Province Hospital of Occupational Diseases, Jinan, China; 4 Department of Oncology, Fengjie County People’s Hospital, Chongqing, China

**Keywords:** ferroptosis, inflammatory cell death, necroptosis, PANoptosis, pyroptosis

## Abstract

Inflammatory cell death has emerged as a critical interface between cancer therapy and antitumor immunity. Unlike relatively non-inflammatory cell death, pyroptosis, necroptosis, ferroptosis, and PANoptosis can generate or release damage-associated molecular patterns (DAMPs), tumor antigens, inflammatory cytokines, oxidized lipids, and nucleic acid ligands that reshape the tumor microenvironment. These death programs link therapeutic stress to dendritic cell activation, antigen cross-presentation, type I interferon signaling, cyclic GMP–AMP synthase (cGAS)–stimulator of interferon genes (STING) signaling, and cytotoxic T-cell responses. However, their immune consequences are highly context dependent. Preclinical studies suggest that selected inflammatory cell death pathways may improve responses to immune checkpoint blockade under defined experimental conditions; however, these effects vary with tumor type, cellular context, and treatment modality, and their clinical relevance remains to be established. Conversely, ferroptosis or PANoptosis in suppressive myeloid cells can reinforce immune evasion. This review summarizes recent experimental advances connecting inflammatory cell death with tumor immunity, focusing on molecular executors, immune mediators, therapeutic opportunities, biomarkers, and translational challenges. We propose that future cancer therapy should move beyond simply inducing tumor cell death toward designing immune-productive death programs that are spatially controlled, cell-type selective, and rationally synchronized with immunotherapy.

## Highlights

- Pyroptosis, necroptosis, ferroptosis, and PANoptosis reshape tumor immunity.
- The immune outcome of inflammatory death depends on the dying cell type.
- Preclinical ICB benefits require tumor-specific and clinical validation.

## 1 Introduction

Therapeutic resistance remains a major barrier to durable cancer control. Although surgery, radiotherapy, chemotherapy, targeted therapy, and immune checkpoint blockade have substantially improved outcomes in selected tumor types, many patients still experience local recurrence, metastatic progression, or incomplete responses (1–4). In the era of cancer immunotherapy, this limitation is particularly evident: only a subset of patients develop durable responses to immune checkpoint inhibitors, while many tumors remain immunologically “cold,” poorly infiltrated by effector T cells, or dominated by suppressive myeloid and stromal compartments. These clinical observations indicate that eliminating tumor cells is not sufficient; the immunological context in which tumor cells die is equally important.

Traditionally, cancer cell death was largely viewed as a terminal cytotoxic event. In this view, the therapeutic goal was to maximize tumor cell killing through DNA damage, mitotic catastrophe, apoptosis, or metabolic collapse (5–9). However, this concept has changed substantially. Accumulating evidence shows that the mode of regulated cell death determines whether dying tumor cells are immunologically silent, tolerogenic, inflammatory, or truly immunogenic (10–12). Tumor cell death can either promote immune activation through the release of tumor antigens, damage-associated molecular patterns (DAMPs), and inflammatory mediators or reinforce immunosuppression by recruiting myeloid-derived suppressor cells, promoting chronic inflammation, or impairing effector lymphocytes. Therefore, the central question is no longer simply “how many tumor cells die,” but rather “how tumor cells die, where they die, and which immune circuits are engaged after death.”

Inflammatory forms of regulated cell death have emerged as key bridges between tumor cytotoxicity and antitumor immunity. Pyroptosis, necroptosis, ferroptosis, and PANoptosis are particularly relevant because they are accompanied by membrane disruption, danger signal release, cytokine production, lipid peroxidation, nucleic acid sensing, or coordinated activation of innate immune pathways (13–17). In this sense, inflammatory cell death represents not only a mechanism of tumor elimination but also a form of immune instruction (18, 19). Recent experimental studies have demonstrated that pyroptosis, necroptosis, ferroptosis, and PANoptosis can influence antitumor immunity through distinct molecular mechanisms, providing potential opportunities for therapeutic intervention (20, 21). Nevertheless, the immune consequences of these death programs depend on the cellular context, the availability of functional immune-sensing pathways, and the interaction between dying cells and the surrounding tumor microenvironment. Importantly, inflammatory cell death does not invariably promote antitumor immunity (22). Its consequences may differ across malignant, stromal, and immune-cell compartments, potentially supporting either immune activation or immunosuppression (23). This context dependence underscores the need to evaluate inflammatory cell death within the broader tumor ecosystem rather than as an isolated molecular event.

In this review, we discuss how pyroptosis, necroptosis, ferroptosis, and PANoptosis connect cancer cell death with antitumor immunity. We first establish a conceptual framework in which inflammatory cell death is viewed as an immune-regulatory circuit involving triggers, executors, immune mediators, and downstream immune outcomes. We then summarize the molecular and immunological features of each death modality, with a particular focus on experimental studies linking these pathways to radiotherapy, chemotherapy, immune checkpoint blockade, natural compounds, and nanomedicine. Finally, we discuss pathway crosstalk, cell-type specificity, therapeutic opportunities, biomarkers, and translational challenges. A deeper understanding of inflammatory cell death may help shift cancer therapy from simply inducing tumor cell death toward designing immune-productive death programs that generate durable systemic antitumor immunity.

## 2 Conceptual framework: inflammatory cell death as an immune-regulatory circuit

### 2.1 From cytotoxic death to immunological death

The immunological consequences of regulated cell death cannot be inferred solely from the extent of tumor cell killing (24). Although apoptosis is often associated with relatively limited inflammation under conditions of efficient efferocytosis, inflammatory death programs can expose or release intracellular components that interact with the surrounding immune microenvironment (25). However, inflammatory signaling does not necessarily establish immunogenic cell death (26). Productive antitumor immunity additionally requires effective antigen presentation, appropriate immune activation, and preservation of functional effector lymphocytes (27, 28). Accordingly, inflammatory cell death should be evaluated not only by its molecular execution pathway but also by the quality of the immune response it generates (29).

A useful way to understand this process is to view inflammatory cell death as a four-step circuit: trigger, executor, immune mediator, and immune outcome. The trigger may involve radiation-induced DNA damage, chemotherapy-induced mitochondrial stress, immune cell-derived interferon-γ (IFN-γ), lipid metabolic rewiring, pattern-recognition receptor activation, reactive oxygen species (ROS) generation during photodynamic therapy, or nanomedicine-delivered stress signals (30–34). The executor is the molecular machinery that commits the cell to a specific death pathway, such as gasdermins in pyroptosis, RIPK3-MLKL in necroptosis, lipid peroxidation machinery in ferroptosis, or PANoptosome-associated proteins in PANoptosis. The immune mediators include DAMPs, cytokines, tumor antigens, mitochondrial DNA, oxidized lipids, and type I interferons. The final immune outcome may involve dendritic cell (DC) maturation, CD8^+^ T-cell priming, increased responsiveness to immune checkpoint blockade, systemic antitumor effects, or, in unfavorable contexts, myeloid-driven immunosuppression.

### 2.2 Core immune outputs of inflammatory cell death

Although pyroptosis, necroptosis, ferroptosis, and PANoptosis differ in their execution mechanisms, they can converge on several immune-regulatory processes, including danger-signal release, innate immune sensing, antigen presentation, and inflammatory remodeling (35, 36). These shared outputs should not be interpreted as functionally equivalent (37). Gasdermin-mediated pyroptosis is characterized by membrane pore formation, necroptosis involves RIPK3–MLKL-dependent membrane disruption, ferroptosis is driven by iron-dependent lipid peroxidation, and PANoptosis coordinates interconnected pyroptotic, apoptotic, and necroptotic machinery (21, 38). Importantly, the generation of inflammatory mediators does not necessarily result in effective dendritic-cell priming or cytotoxic T-cell responses (29). The immunological outcome depends on the molecular execution pathway and the capacity of the surrounding immune microenvironment to respond appropriately (39).

### 2.3 Cell-type specificity: tumor cells versus immune cells

A central principle in this field is that the immune consequence of inflammatory cell death depends on the identity of the dying cell. When inflammatory death occurs in tumor cells, it may enhance antitumor immunity by releasing tumor antigens and danger signals into an immune-competent microenvironment. For example, GSDME-mediated pyroptosis induced by radiotherapy or chemotherapy can promote immune activation and T-cell infiltration (18, 40). RIPK3-dependent necroptosis can enhance dendritic cell cross-presentation and support systemic antitumor responses (19). CD8^+^ T-cell-driven ferroptosis in tumor cells provides an additional example in which immune effector cells actively induce a metabolic death program in malignant cells (41).

By contrast, inflammatory death in immune cells may produce different or even opposing effects. Ferroptosis of tumor-associated neutrophils can promote immune suppression rather than tumor clearance (22). PANoptosis-associated programs in tumor-associated neutrophils have also been linked to an immunosuppressive microenvironment in non-small cell lung cancer (23). These findings caution against a simplistic therapeutic strategy of globally inducing inflammatory cell death. Instead, future approaches should aim to induce immune-productive death selectively in malignant cells while preserving or enhancing the function of dendritic cells, cytotoxic T cells, natural killer cells, and other antitumor immune populations.

### 2.4 Cellular context determines the immune outcome of inflammatory cell death

The identity of the dying cell and the functional state of the surrounding immune microenvironment are critical determinants of inflammatory cell death outcomes. Death occurring in malignant cells may facilitate immune recognition, whereas death-associated processes in myeloid or effector lymphocyte compartments can generate suppressive or functionally detrimental consequences (19–21, 42, 43). The magnitude, duration, and spatial distribution of inflammatory signaling further influence whether local cell death contributes to productive antitumor immunity or persistent tumor-promoting inflammation. These considerations establish cellular specificity and immune competence as essential dimensions of inflammatory cell death, which are examined in greater detail in Section 8. **Table 1** summarizes the major molecular features and immune consequences of pyroptosis, necroptosis, ferroptosis, and PANoptosis in cancer. **Figure 1** provides the conceptual framework of the review by illustrating how different inflammatory cell death programs connect therapeutic stress to innate sensing, antigen presentation, and T-cell-mediated antitumor immunity. It also highlights that the final immune outcome depends on both the death modality and the cellular context.

**Table 1:** Molecular executors and immune consequences of inflammatory cell death in cancer.

Death modality	Key executors or regulators	Major immune mediators	Main immune consequence	Representative evidence	References
Pyroptosis	GSDME, GSDMD, caspase-3, caspase-1	ATP, HMGB1, IL-1β, IL-18, tumor antigens	DC activation, CD8^+^ T-cell infiltration, enhanced ICB response	Ionizing radiation induces GSDME-mediated pyroptosis and antitumor immunity	(18)
Pyroptosis	GSDME, caspase-3	ATP, HMGB1, phagocytosis-associated signals	Chemotherapy-induced immune activation	Neoadjuvant chemotherapy promotes GSDME-mediated pyroptosis in breast cancer	(40)
Pyroptosis regulation	CDC20, GSDME	DAMPs, T-cell inflammatory signals	Increased anti-PD-1 sensitivity	CDC20 inhibition facilitates GSDME-mediated pyroptosis in prostate cancer	(44)
Engineered pyroptosis	Gasdermin delivery, ESCRT inhibition	DAMPs, inflammatory cell recruitment	Amplified pyroptosis and stronger antitumor immunity	Blocking ESCRT-dependent membrane repair enhances gasdermin-induced tumor pyroptosis	(45)
Necroptosis	RIPK1, RIPK3, MLKL	mtDNA, DAMPs, cGAS-STING, IFN-I	DC cross-presentation, abscopal immunity	Cisplatin induces RIPK3-dependent necroptosis and enhances radiation-induced abscopal effects	(19)
Necroptosis	RIPK3, MLKL	IFN-I, inflammatory mediators	Protective antitumor immunity	Necroptosis stimulates interferon-mediated antitumor immunity	(46)
Ferroptosis in tumor cells	ACSL4, GPX4, SLC7A11, lipid ROS	Lipid peroxidation signals, IFN-γ-associated metabolic stress	CD8^+^ T-cell-mediated tumor killing	CD8^+^ T cells and fatty acids induce ACSL4-dependent tumor ferroptosis	(41)
Ferroptosis in immune cells	Ferroptotic tumor neutrophils, PMN-MDSCs	Oxidized lipids, suppressive inflammatory mediators	Immune suppression and T-cell dysfunction	Ferroptosis of tumor neutrophils causes immune suppression	(22)
PANoptosis	ZBP1, RIPK3, caspase-8, caspase-1, gasdermins, MLKL	DAMPs, cytokines, STING-related signals	Integrated inflammatory death and immune remodeling	PDT-induced PANoptosis enhances antitumor immunity with LAG3 blockade	(21)
PANoptosis in suppressive niches	TAN-associated PANoptosis programs	HMGB1-related inflammatory signals, checkpoint-associated pathways	Immunosuppressive TME remodeling	TAN-associated PANoptosis fosters an immunosuppressive NSCLC microenvironment	(23)

ACSL4, acyl-CoA synthetase long-chain family member 4; ATP, adenosine triphosphate; DC, dendritic cell; ESCRT, endosomal sorting complexes required for transport; GSDMD, gasdermin D; GSDME, gasdermin E; HMGB1, high-mobility group box 1; ICB, immune checkpoint blockade; IFN-I, type I interferon; MLKL, mixed lineage kinase domain-like protein; RIPK3, receptor-interacting protein kinase 3.

**Figure 1:**
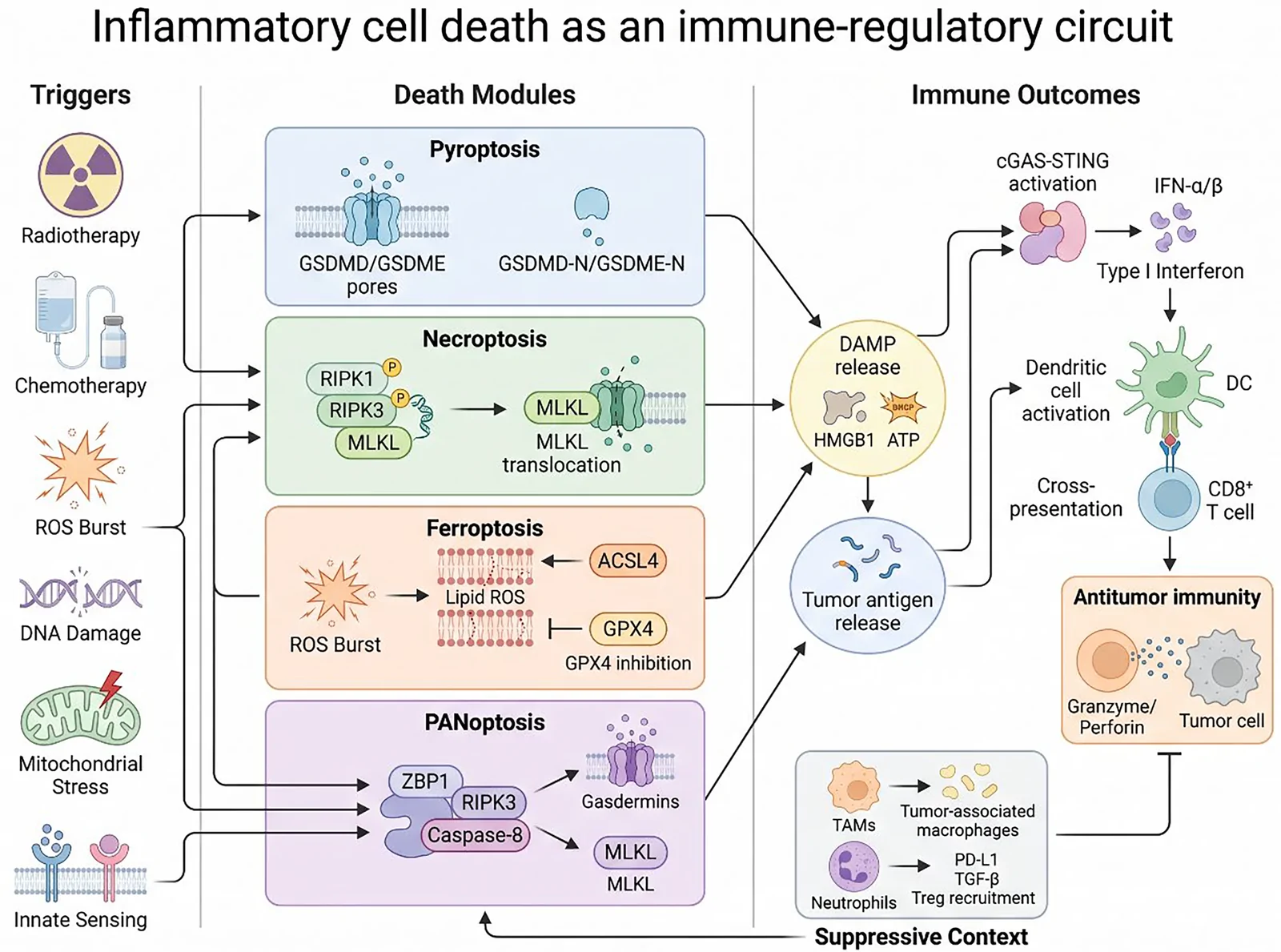
Conceptual overview of inflammatory cell death in cancer. Pyroptosis, necroptosis, ferroptosis, and PANoptosis are triggered by therapeutic and stress signals and converge on shared immune outputs, including DAMP release, antigen presentation, type I interferon signaling, and CD8^+^ T-cell activation. Depending on cellular context, these pathways may drive either productive antitumor immunity or immunosuppressive inflammation.

## 3 Pyroptosis in cancer immunity: gasdermin pores as immune-activating gates

### 3.1 Molecular basis of tumor pyroptosis

Pyroptosis is a lytic and inflammatory form of regulated cell death characterized by gasdermin activation, membrane pore formation, cell swelling, and release of intracellular contents (42, 47, 48). The gasdermin family functions as the key executioner of pyroptosis. After proteolytic cleavage by inflammatory caspases or apoptotic caspases, the N-terminal fragment of gasdermins oligomerizes and inserts into the plasma membrane to form pores. These pores disrupt ionic gradients, allow the release of inflammatory mediators, and ultimately lead to cell rupture. In immune cells, canonical pyroptosis is often associated with inflammasome activation, caspase-1-mediated cleavage of gasdermin D (GSDMD), and maturation of interleukin-1β (IL-1β) and interleukin-18 (IL-18). In tumor cells, however, therapy-induced pyroptosis frequently involves caspase-3-mediated cleavage of gasdermin E (GSDME) downstream of DNA damage, mitochondrial stress, or chemotherapy-induced apoptosis-like signaling (43, 49–52).

GSDME-mediated pyroptosis is particularly relevant in cancer because it can convert a classically apoptotic stimulus into an inflammatory form of tumor cell death. When GSDME expression is sufficient, caspase-3 activation does not only drive apoptotic dismantling but also cleaves GSDME, allowing the N-terminal fragment to form membrane pores (13, 14, 53–55). This switch changes the immunological nature of tumor cell death. Instead of being rapidly cleared as apoptotic bodies, pyroptotic tumor cells release DAMPs, tumor antigens, and inflammatory signals that can activate dendritic cells and recruit cytotoxic lymphocytes. Therefore, GSDME expression may determine whether a cytotoxic therapy produces immunologically silent killing or immune-stimulatory tumor destruction. However, pyroptosis is not simply an “on-off” event. The strength and duration of gasdermin pore formation, the efficiency of membrane repair, the abundance of DAMPs, and the immune composition of the tumor microenvironment all influence the final immune outcome. In this regard, pyroptosis can be viewed as a controllable immune gate: gasdermin pores open the dying tumor cell to immune recognition, but the downstream response depends on whether antigen-presenting cells and effector T cells can convert this inflammatory signal into productive antitumor immunity.

### 3.2 Radiotherapy-induced pyroptosis and antitumor immunity

Radiotherapy is one of the most important therapeutic triggers of inflammatory tumor cell death (56). In addition to causing DNA double-strand breaks and mitotic catastrophe, ionizing radiation can reshape the tumor immune microenvironment through antigen release, DAMP exposure, and innate immune activation (57, 58). Recent evidence indicates that GSDME-mediated pyroptosis is one mechanism by which radiotherapy converts local tumor damage into antitumor immune activation (18).

Ionizing radiation has been shown to induce GSDME-mediated pyroptosis in tumor cells and thereby enhance antitumor immunity (18). Mechanistically, radiation-induced DNA damage and stress signaling can activate caspase-3, leading to GSDME cleavage and pyroptotic membrane disruption. This process promotes the release of inflammatory signals from dying tumor cells and increases CD8^+^ T-cell infiltration within the tumor microenvironment. Importantly, the immune effect of radiotherapy is therefore not limited to direct tumor cell killing. Instead, radiation-induced pyroptosis can act as an *in situ* vaccination-like event, increasing the visibility of tumor antigens to the immune system and supporting effector T-cell-mediated tumor control. This finding has several implications. First, GSDME status may influence the immune responsiveness of radiotherapy-treated tumors. Tumors with higher GSDME expression may be more likely to undergo pyroptotic conversion after radiation, whereas GSDME-silenced tumors may die through less inflammatory routes (59–63). Second, radiotherapy-induced pyroptosis provides a mechanistic rationale for combining radiotherapy with immune checkpoint blockade. By increasing antigen availability and T-cell recruitment, pyroptosis may help overcome pre-existing immune exclusion or weak T-cell priming. Third, pyroptosis may contribute to systemic immune effects after local therapy, especially when combined with agents that enhance dendritic cell activation or prevent T-cell exhaustion.

Nevertheless, radiotherapy-induced pyroptosis must be interpreted carefully. Excessive inflammatory injury may also damage normal tissues, and tumor heterogeneity may limit uniform pyroptotic responses. Therefore, future studies should clarify how radiation dose, fractionation, tumor GSDME expression, DNA damage response status, and immune contexture jointly determine whether radiotherapy-induced pyroptosis produces effective antitumor immunity.

### 3.3 Chemotherapy-induced GSDME pyroptosis

Chemotherapy remains a major trigger of therapy-induced tumor cell death. Although many chemotherapeutic agents were originally developed as cytotoxic drugs, their immune effects are increasingly recognized. Some agents can induce immunogenic cell death, enhance antigen release, or increase immune cell infiltration. GSDME-mediated pyroptosis provides one mechanistic explanation for how chemotherapy can transform tumor cell killing into immune activation (64–66).

In breast cancer, neoadjuvant chemotherapy has been reported to promote GSDME-mediated pyroptosis and antitumor immunity (40). This study is important because it links a clinically relevant treatment setting with pyroptotic tumor cell death and immune remodeling. Chemotherapy-induced GSDME pyroptosis was associated with the release of ATP and HMGB1, enhanced macrophage phagocytosis, and increased T-cell-associated immune responses. These observations suggest that chemotherapy may not only reduce tumor burden directly but also generate an inflammatory microenvironment that facilitates immune recognition. The chemotherapy-pyroptosis axis is particularly relevant for tumors with partial immune responsiveness. In such tumors, chemotherapy-induced pyroptosis may provide the initial inflammatory signal required for dendritic cell recruitment and antigen presentation. Once T cells are primed and recruited, immune checkpoint blockade may further sustain their cytotoxic activity. Therefore, GSDME-mediated pyroptosis may represent a mechanistic bridge between conventional chemotherapy and modern immunotherapy (67–71).

However, several questions remain unresolved. First, GSDME is frequently epigenetically silenced in cancers, which may limit pyroptotic conversion after chemotherapy. Second, different chemotherapeutic agents may vary in their ability to activate caspase-3 and cleave GSDME. Third, the same inflammatory signals that activate antitumor immunity may also recruit suppressive myeloid populations under certain conditions. Thus, the therapeutic goal should not be chemotherapy-induced inflammation alone, but chemotherapy-induced immune-productive pyroptosis.

### 3.4 Upstream regulators of pyroptosis: from CDC20 to natural compounds

Pyroptosis is tightly regulated by upstream molecular circuits that determine gasdermin expression, cleavage, stability, and downstream immune signaling. Identifying these regulators is important because they may serve as biomarkers or therapeutic targets for enhancing pyroptosis-mediated antitumor immunity (36). CDC20 has been identified as a regulator of GSDME-mediated pyroptosis in prostate cancer. In a preclinical study, CDC20 inhibition increased GSDME abundance and promoted pyroptotic cell death, while combination treatment with anti-PD-1 antibodies improved antitumor responses in immunocompetent mouse models (44). Similarly, curcumin enhanced caspase-3/GSDME-dependent pyroptosis and increased the antitumor efficacy of PD-1 blockade in experimental colorectal cancer models (72). These findings provide mechanistic support for combining pyroptosis induction with immune checkpoint blockade. However, the observed benefits remain restricted to the experimental models investigated and should not be interpreted as evidence of clinical efficacy. Differences in GSDME expression, tumor immunogenicity, drug exposure, and immune-cell composition may substantially influence treatment responses. Clinical studies are therefore required to determine whether these combinations provide therapeutic benefits in patients.

Together, these studies suggest that pyroptosis can be therapeutically tuned at multiple levels. One strategy is to increase gasdermin expression or prevent its degradation. Another is to enhance caspase-mediated gasdermin cleavage. A third is to combine pyroptosis-inducing agents with immunotherapy to sustain the effector phase of antitumor immunity. However, the safety of systemic pyroptosis induction remains a major concern. Because gasdermin activation can cause strong inflammation, future strategies should prioritize tumor-selective delivery, biomarker-guided patient selection, and rational combination timing.

### 3.5 Engineering pyroptosis intensity: overcoming membrane repair

An emerging concept is that the magnitude of pyroptosis is not determined solely by gasdermin activation. Once gasdermin pores form in the plasma membrane, cells may activate membrane repair mechanisms to limit lytic death. The endosomal sorting complexes required for transport (ESCRT) machinery can facilitate plasma membrane repair by removing damaged membrane regions, thereby limiting the consequences of gasdermin-mediated pore formation. Therefore, tumor cells may partially escape pyroptosis by repairing gasdermin-induced membrane injury.

Preventing ESCRT-dependent membrane repair has been shown to enhance gasdermin-induced tumor pyroptosis and augment antitumor immune responses (45). This work is important because it shifts attention from pyroptosis initiation to pyroptosis execution intensity. In other words, it is not enough to activate gasdermins; the durability of pore formation and the failure of membrane repair may determine whether tumor cells undergo full inflammatory lysis. By engineering strategies that enhance gasdermin pore activity or block membrane repair, it may be possible to amplify DAMP release, antigen exposure, and immune cell recruitment. This approach also highlights the potential of synthetic biology and nanomedicine in controlling inflammatory cell death. Direct delivery of pyroptosis executors, tumor-targeted activation of gasdermins, or combination with membrane repair inhibitors could provide more precise control over pyroptotic intensity (66, 73–76). Such strategies may be particularly useful in tumors that are resistant to conventional apoptosis or have weak immunogenicity. However, excessive pyroptosis could also cause local tissue damage or systemic inflammation. Therefore, engineering pyroptosis requires a balance between sufficient inflammatory signaling and acceptable safety.

Collectively, these findings identify gasdermin activation and membrane-repair capacity as important determinants of therapeutic pyroptosis. **Figure 2** summarizes the major mechanisms through which radiotherapy, chemotherapy, and selected experimental modulators regulate GSDME-mediated tumor-cell pyroptosis and its associated immune effects.

**Figure 2:**
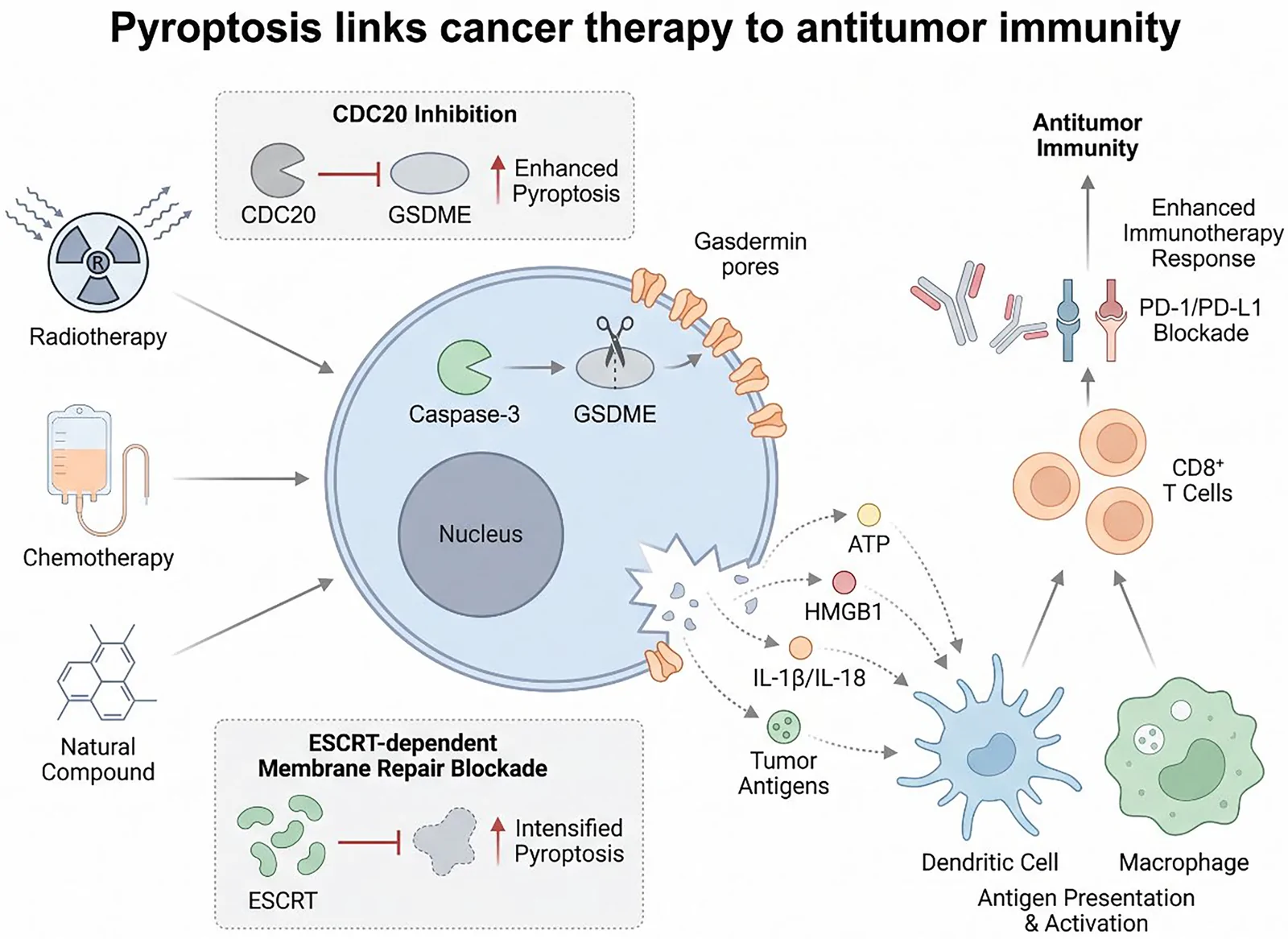
Therapeutic induction of tumor pyroptosis promotes antitumor immunity. Radiotherapy, chemotherapy, and selected modulators activate caspase-3–GSDME signaling, leading to gasdermin pore formation and lytic tumor cell death. Pyroptosis releases DAMPs and tumor antigens, enhances phagocyte and dendritic cell activation, recruits CD8^+^ T cells, and can improve sensitivity to immune checkpoint blockade.

## 4 Necroptosis in cancer: RIPK3–MLKL signaling, type I interferon, and immunogenicity

### 4.1 Molecular basis of necroptosis

Necroptosis is a regulated form of lytic cell death that morphologically resembles necrosis but is controlled by defined signaling molecules (77–79). Unlike apoptosis, which usually preserves plasma membrane integrity and is often immunologically silent when apoptotic bodies are rapidly cleared, necroptosis culminates in membrane rupture, intracellular content release, and inflammatory signaling. This makes necroptosis particularly relevant to cancer immunity, where the immunological consequence of tumor cell death may be as important as the extent of tumor cell killing.

The canonical necroptotic pathway is mainly governed by receptor-interacting protein kinase 1 (RIPK1), receptor-interacting protein kinase 3 (RIPK3), and mixed lineage kinase domain-like protein (MLKL) (78, 80–83). Under conditions in which death receptor signaling is activated and caspase-8 activity is insufficient or inhibited, RIPK1 and RIPK3 can assemble into a necrosome complex (84). RIPK3 then phosphorylates MLKL, leading to MLKL oligomerization, translocation to the plasma membrane, membrane permeabilization, and eventual cell rupture (85). This process releases DAMPs, nucleic acids, mitochondrial components, and other intracellular danger signals into the tumor microenvironment. In cancer, necroptosis has a dual biological meaning. On one hand, it can overcome apoptosis resistance, which is a common feature of malignant cells exposed to chemotherapy, radiotherapy, or targeted therapy. On the other hand, necroptotic tumor cells can become an inflammatory source of antigens and innate immune ligands (35). Through DAMP release and nucleic acid sensing, necroptosis may promote dendritic cell maturation, antigen cross-presentation, type I interferon production, and T-cell-mediated antitumor immunity (19). Therefore, necroptosis is not only a backup death mechanism when apoptosis fails but also a potential immunogenic death program that can be therapeutically exploited (39).

However, necroptosis must also be interpreted in a context-dependent manner. Persistent or poorly controlled necroptotic inflammation may support tumor-promoting inflammation, tissue damage, angiogenesis, or suppressive myeloid recruitment. Thus, as with other inflammatory cell death modalities, the goal is not simply to maximize necroptosis but to induce spatially and temporally controlled necroptosis that favors productive antitumor immunity.

### 4.2 Cisplatin-induced necroptosis and the abscopal effect

Cisplatin is classically viewed as a DNA-damaging chemotherapeutic agent. However, recent evidence suggests that its therapeutic relevance may extend beyond direct cytotoxicity. Cisplatin can induce RIPK3-dependent necroptosis in tumor cells and thereby enhance the immunogenicity of local tumor treatment (19). This finding is important because it connects a conventional chemotherapeutic drug with innate immune sensing and systemic antitumor responses.

Mechanistically, cisplatin-induced necroptosis promotes the release of mitochondrial DNA from dying tumor cells. Mitochondrial DNA can function as a danger-associated nucleic acid signal and activate the cGAS-STING pathway (86–88). Activation of cGAS-STING subsequently drives type I interferon responses, which are essential for dendritic cell activation and antigen cross-presentation. Through this pathway, necroptotic tumor cells can help convert local cytotoxic stress into adaptive immune priming. This mechanism is particularly relevant to the radiation-induced abscopal effect. The abscopal effect refers to regression of non-irradiated lesions after local radiotherapy, a phenomenon generally believed to depend on systemic antitumor immunity. By inducing RIPK3-dependent necroptosis and activating mitochondrial DNA–cGAS–STING signaling, cisplatin can enhance dendritic cell cross-presentation and T-cell-mediated immune responses, thereby strengthening radiation-induced systemic tumor control (19). This provides a mechanistic rationale for combining DNA-damaging chemotherapy, radiotherapy, and immunotherapy.

The cisplatin-necroptosis axis also illustrates a broader principle: some conventional therapies may be reinterpreted as immunological modulators. Their efficacy may depend not only on tumor cell-intrinsic sensitivity to DNA damage but also on whether the induced death program generates immune-stimulatory signals. Therefore, markers such as RIPK3, MLKL, cGAS-STING activity, type I interferon signatures, and dendritic cell activation may help identify tumors in which chemotherapy-induced necroptosis is more likely to support antitumor immunity.

### 4.3 Type I interferon-mediated immunity in necroptosis

Although necroptosis is often described in terms of DAMP release, its immunological output is not limited to passive leakage of intracellular components. Necroptosis can actively promote type I interferon-mediated protective antitumor immunity (46). This concept shifts the understanding of necroptosis from a purely lytic death mode to a death-associated immune signaling platform. Type I interferons play a central role in cancer immune surveillance. They support dendritic cell maturation, antigen cross-presentation, cytotoxic T-cell priming, natural killer cell activation, and the development of systemic antitumor immunity. When necroptotic death engages type I interferon pathways, dying tumor cells may act as an endogenous adjuvant source. In this setting, RIPK3-dependent necroptosis can help establish an immune-activated tumor microenvironment that favors protective immunity rather than silent clearance.

The necroptosis–type I interferon–dendritic cell–T-cell signaling axis provides a conceptual framework for understanding how necroptotic signaling may contribute to adaptive antitumor immunity. First, necroptotic tumor cells undergo MLKL-mediated membrane disruption and release intracellular danger signals. Second, these signals activate innate immune pathways in tumor cells or surrounding antigen-presenting cells. Third, type I interferon production enhances dendritic cell function and antigen presentation. Finally, primed T cells mediate tumor cell killing and may amplify secondary waves of immunogenic death. This feedback loop provides a plausible explanation for how necroptosis can transform local tumor injury into systemic immune protection. This mechanism also has therapeutic implications. Tumors with defective type I interferon signaling may fail to convert necroptosis into effective antitumor immunity, even if necroptotic death occurs (89–93). Conversely, tumors with intact innate immune sensing may be more responsive to necroptosis-inducing strategies. Therefore, future therapeutic designs should consider not only whether necroptosis is induced, but whether the downstream IFN-I and antigen-presentation machinery remains functional.

### 4.4 Engineering necroptosis for cancer vaccines and cell-based immunotherapy

The immunogenic nature of necroptosis has inspired engineering strategies for cancer vaccines and cell-based therapies. One attractive approach is to build inducible necroptotic systems that can be activated in a controlled manner. Such systems may serve two purposes: they can function as safety switches to eliminate engineered cells when needed, and they can simultaneously generate immunogenic cell death to enhance antitumor immune responses.

An inducible RIPK3-driven necroptotic system has been developed to enhance cancer cell-based immunotherapy while improving safety (94). This strategy takes advantage of the central role of RIPK3 in necroptosis execution. By engineering cells to undergo RIPK3-driven necroptosis upon induction, it becomes possible to trigger inflammatory death in a predictable and controllable manner. In the context of cancer vaccines, necroptotic tumor cells may provide both tumor antigens and endogenous adjuvant signals, thereby improving dendritic cell activation and T-cell priming. This approach has several conceptual advantages. First, necroptotic cancer cells may be more immunogenic than apoptotic cancer cells because they release DAMPs and inflammatory mediators (77, 95–98). Second, inducibility allows better control over timing and intensity, which may reduce the risk of uncontrolled inflammation. Third, the system may be incorporated into cell-based immunotherapies as a safeguard, allowing therapeutic cells to be eliminated if severe toxicity or off-target effects occur. Fourth, necroptotic death may enhance antigen spreading, enabling immune recognition of a broader repertoire of tumor antigens.

Nevertheless, engineered necroptosis also faces translational challenges. The inflammatory intensity must be carefully tuned, because excessive necroptosis could cause local tissue damage or systemic inflammatory toxicity. The engineered system must be stable, controllable, and compatible with clinical manufacturing. Moreover, the immune microenvironment must be permissive; necroptotic vaccines may be less effective in tumors dominated by suppressive myeloid cells, exhausted T cells, or defective antigen-presenting cells. Thus, engineered necroptosis may be most effective when combined with immune checkpoint blockade, dendritic cell activation, or myeloid reprogramming strategies.

### 4.5 Preclinical evidence for combining necroptosis induction with immune checkpoint blockade

Pharmacological induction or enhancement of necroptosis is emerging as a potential strategy to improve immune checkpoint blockade. Immune checkpoint inhibitors require pre-existing or therapy-induced antitumor immune responses. Tumors that lack antigen presentation, T-cell infiltration, or inflammatory priming often respond poorly. Necroptosis may help convert such immune-cold tumors into more inflamed and immune-responsive tumors by releasing DAMPs, activating innate immune sensing, and promoting T-cell recruitment (99–103).

Preclinical evidence suggests that pharmacological modulation of necroptosis may improve responses to immune checkpoint blockade in selected tumor models (39). For example, MDM2 inhibition has been shown to stabilize RIPK3, enhance necroptotic signaling, and improve the antitumor efficacy of anti-PD-1 therapy in experimental models (104). These findings suggest that necroptosis-associated inflammatory signaling may help overcome insufficient immune activation under specific conditions. Nevertheless, the therapeutic interaction has not been established as clinically effective, and its relevance may depend on functional RIPK3–MLKL machinery, intact innate immune sensing, and a permissive T-cell microenvironment. Furthermore, the antitumor activity of MDM2 inhibitors may involve biological effects beyond necroptosis (105). Future studies should determine the relative contribution of necroptotic signaling to combination efficacy and evaluate whether these findings can be reproduced across tumor types and clinically relevant treatment settings.

These findings suggest that successful necroptosis-based therapy requires both functional RIPK3–MLKL signaling and an immune microenvironment capable of responding to death-associated signals. **Figure 3** illustrates the RIPK3–MLKL pathway and the potential contribution of mitochondrial DNA–cGAS–STING signaling to downstream antitumor immune responses.

**Figure 3:**
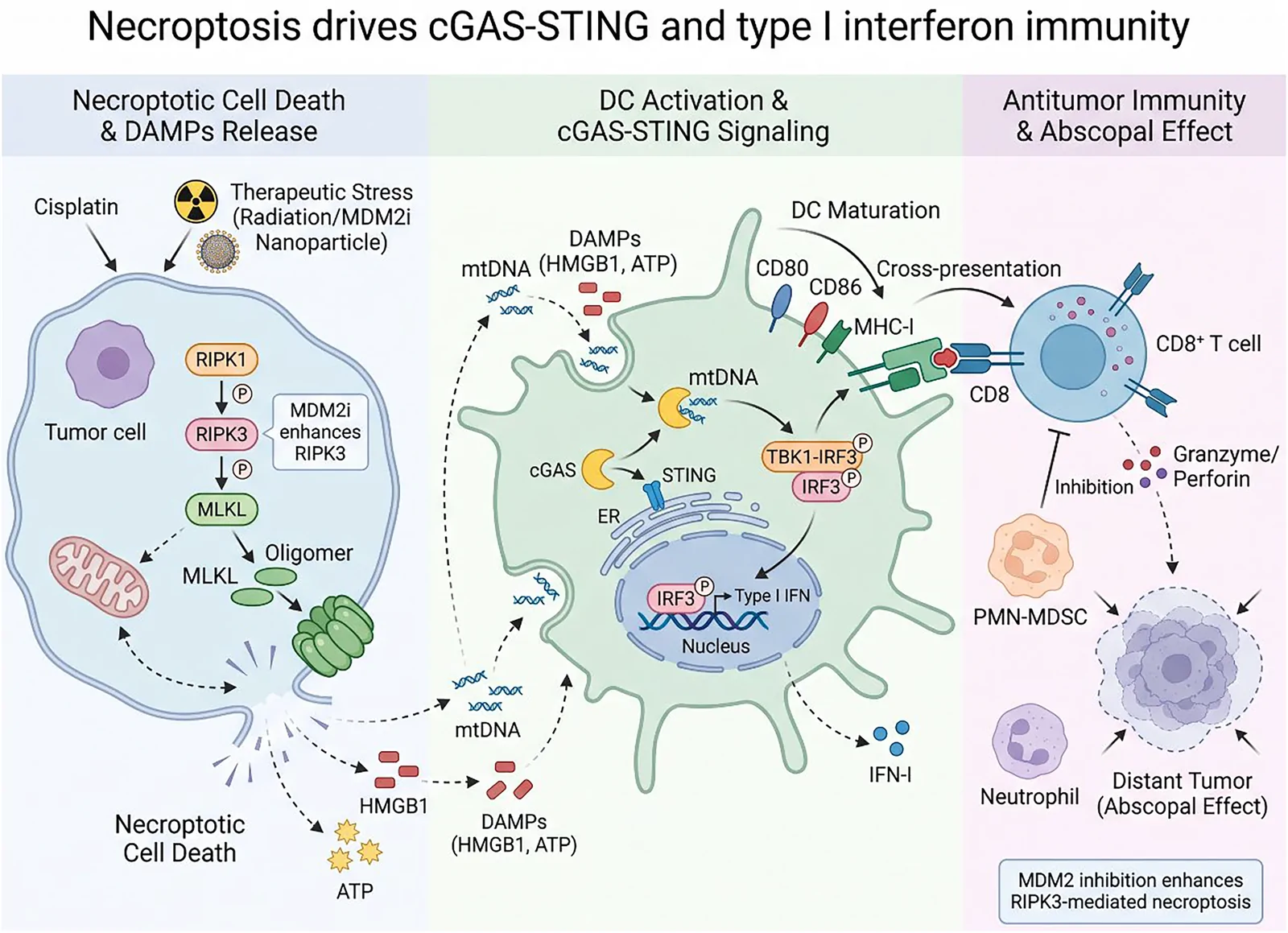
Necroptosis converts tumor cell death into innate and adaptive immune activation. Therapeutic stress induces RIPK1–RIPK3–MLKL signaling, leading to membrane rupture and release of mitochondrial DNA and DAMPs. These signals activate cGAS-STING and type I interferon pathways, promote dendritic cell cross-presentation, stimulate CD8^+^ T-cell responses, and may enhance systemic antitumor effects.

## 5 Ferroptosis in antitumor immunity: a context-dependent inflammatory death program

### 5.1 Molecular basis of ferroptosis

Ferroptosis is an iron-dependent form of regulated cell death driven by excessive lipid peroxidation. Unlike pyroptosis and necroptosis, ferroptosis is not primarily defined by gasdermin pore formation or MLKL-mediated membrane rupture (82, 106, 107). Instead, it is characterized by the accumulation of phospholipid hydroperoxides, failure of antioxidant defense systems, iron-catalyzed oxidative stress, and membrane lipid damage. This makes ferroptosis a metabolically controlled death program that links redox biology, lipid metabolism, iron homeostasis, and tumor immunity.

Several molecular modules determine ferroptosis sensitivity. The cystine/glutamate antiporter system x_c^-^, which contains solute carrier family 7 member 11 (SLC7A11) as its transporter subunit, mediates cystine uptake to support glutathione synthesis. Glutathione is required for the activity of glutathione peroxidase 4 (GPX4), a central enzyme that detoxifies lipid hydroperoxides (108–113). When SLC7A11 is inhibited or GPX4 activity is impaired, lipid peroxides accumulate and drive ferroptotic death. Acyl-CoA synthetase long-chain family member 4 (ACSL4) promotes the incorporation of polyunsaturated fatty acids into phospholipids, thereby increasing the pool of oxidizable membrane lipids. Iron availability and ROS generation further amplify lipid peroxidation.

Although ferroptosis is not a classical cytokine-driven inflammatory death program, it can profoundly influence tumor immunity. Ferroptotic tumor cells may release DAMPs and oxidized lipid signals that affect dendritic cells, macrophages, neutrophils, and T cells. Ferroptosis can also be induced by immune effector mechanisms, especially IFN-γ produced by CD8^+^ T cells. Therefore, ferroptosis should be viewed as a context-dependent immune-metabolic death modality. It may support tumor elimination when it occurs in malignant cells, but it may impair antitumor immunity when it occurs in effector lymphocytes or generates suppressive myeloid programs.

### 5.2 CD8^+^ T cell-induced tumor ferroptosis

One of the most important advances in this field is the recognition that ferroptosis can be directly linked to cytotoxic T-cell immunity. CD8^+^ T cells and fatty acids have been shown to orchestrate tumor ferroptosis and immunity through ACSL4 (41). This study provides strong evidence that ferroptosis is not merely a pharmacologically induced vulnerability but can function as part of endogenous immune-mediated tumor control.

Mechanistically, CD8^+^ T cells produce IFN-γ, which can alter tumor cell metabolism and sensitize malignant cells to ferroptosis. In parallel, fatty acid metabolism contributes to ACSL4-dependent enrichment of polyunsaturated phospholipids, creating a lipid environment that is vulnerable to peroxidation (114–117). The convergence of immune-derived IFN-γ and tumor lipid metabolic state promotes ferroptotic death in cancer cells (118–120). This places ferroptosis downstream of adaptive immunity and suggests that T-cell activation may kill tumor cells not only through perforin/granzyme or death receptor pathways but also by inducing metabolic catastrophe. This finding has several implications for immunotherapy. First, ferroptosis sensitivity may influence the effectiveness of immune checkpoint blockade, because reinvigorated CD8^+^ T cells may rely partly on ferroptosis-inducing mechanisms. Second, tumor cells that upregulate anti-ferroptotic defenses such as SLC7A11 or GPX4 may resist immune-mediated killing. Third, metabolic interventions that increase ACSL4 activity or lipid peroxidation may synergize with T-cell-based immunotherapy (121–124). Finally, ferroptosis biomarkers could help identify tumors that are more likely to respond to immune activation.

However, the therapeutic induction of ferroptosis must be carefully designed. If ferroptosis occurs predominantly in tumor cells, it may enhance tumor control. If it damages CD8^+^ T cells or other immune effector cells, it may weaken immunity. Therefore, the spatial and cellular distribution of ferroptosis is a critical determinant of therapeutic outcome.

### 5.3 Radiotherapy, DNA repair inhibition, and cGAS-linked ferroptosis

Radiotherapy and DNA damage response-targeting agents can also engage ferroptosis-associated immune pathways. In a preclinical colorectal cancer study, inhibition of poly(ADP-ribose) polymerase (PARP) was shown to enhance radiotherapy-induced ferroptosis and antitumor immune responses through cGAS-associated signaling (125). This finding connects DNA damage, innate immune sensing, ferroptosis, and adaptive immunity into a single therapeutic axis.

Radiotherapy generates DNA damage, ROS, and oxidative stress, all of which can contribute to lipid peroxidation. PARP inhibition further increases DNA damage burden and may amplify cytosolic DNA accumulation. Cytosolic DNA can activate cGAS, leading to STING pathway activation and downstream type I interferon responses. In this context, ferroptosis is not isolated from innate immunity; instead, DNA damage-induced cGAS signaling can cooperate with ferroptotic stress to enhance antitumor immune responses (126–130). The DNA damage–cGAS–ferroptosis–CD8^+^ T-cell axis provides a mechanistic rationale for combining radiotherapy, PARP inhibitors, ferroptosis-inducing strategies, and immune checkpoint blockade. Radiotherapy may initiate local tumor injury and oxidative stress; PARP inhibition may amplify DNA damage and cGAS activation; ferroptosis may increase tumor vulnerability and inflammatory signaling; and T-cell immunity may consolidate systemic tumor control. This integrated model is particularly relevant for tumors with limited baseline immune infiltration, where therapy-induced innate immune activation may be required to initiate effective responses.

Nevertheless, several translational questions remain. It is unclear which radiation dose and fractionation schedule most effectively promotes ferroptosis-associated immunity. PARP inhibitors may have different immunological effects depending on tumor DNA repair status and immune context. In addition, ferroptosis induction may interact with normal tissue radiosensitivity. Therefore, future studies should define optimal timing, biomarkers, and safety windows for combining DNA damage therapy with ferroptosis-based immune modulation.

### 5.4 Metabolic regulators of ferroptosis and immune response

Ferroptosis is highly dependent on metabolic state. Tumor cells adapt their lipid metabolism, mitochondrial function, iron handling, and antioxidant systems to survive under oxidative stress. These metabolic adaptations influence not only ferroptosis sensitivity but also the immune composition of the tumor microenvironment. Apolipoprotein L3 (APOL3) has been reported to enhance CD8^+^ T-cell antitumor immunity in colorectal cancer through a mechanism involving lactate dehydrogenase A (LDHA) and ferroptosis (131). This finding suggests that metabolic enzymes traditionally associated with glycolysis or lipid handling may also influence ferroptotic vulnerability and immune responsiveness. By linking APOL3, LDHA, ferroptosis, and CD8^+^ T-cell immunity, this study supports the idea that metabolic regulation of ferroptosis can reshape antitumor immune surveillance. Another example is carnitine palmitoyltransferase 1A (CPT1A), a key enzyme involved in mitochondrial fatty acid oxidation. In lung cancer, CPT1A-mediated metabolic remodeling has been implicated in ferroptosis resistance and impaired antitumor immunity. Experimental targeting of CPT1A increased tumor ferroptosis and improved the response to immune checkpoint blockade in tumor-bearing mice. Although this study also incorporated analyses of human lung cancer samples, the therapeutic benefit of CPT1A inhibition combined with checkpoint blockade was demonstrated primarily in preclinical models. Therefore, these findings support further investigation of CPT1A as a potential therapeutic target but do not establish its clinical efficacy as an immunotherapy sensitizer.

These studies indicate that ferroptosis is deeply embedded in tumor metabolic networks. Therapeutic strategies may target SLC7A11, GPX4, ACSL4, LDHA, CPT1A, iron metabolism, or lipid remodeling enzymes to increase tumor ferroptosis. However, metabolic regulators are rarely tumor-specific. Many immune cells also depend on lipid metabolism and redox balance. Therefore, ferroptosis-based metabolic therapy must avoid compromising effector T-cell survival and function. Ideally, future approaches should exploit metabolic dependencies that are selectively enriched in tumor cells or suppressive stromal compartments while preserving antitumor immune cells.

### 5.5 Ferroptosis heterogeneity and immunotherapy combinations

Ferroptosis sensitivity is heterogeneous across tumor types, tumor subclones, and microenvironmental niches. This heterogeneity has important implications for immunotherapy combinations. In triple-negative breast cancer, ferroptosis heterogeneity has revealed potential strategies for combining ferroptosis induction with immunotherapy (132). This suggests that ferroptosis should not be treated as a uniform vulnerability across all tumors.

Several factors may contribute to ferroptosis heterogeneity. Tumor cells differ in the expression of ferroptosis-related regulators, including SLC7A11, GPX4, ACSL4, ferroptosis suppressor protein 1 (FSP1), iron-handling proteins, lipid desaturases, and antioxidant pathway components (109, 111, 133–135). Hypoxia, nutrient availability, stromal interactions, and immune pressure can further shape ferroptotic susceptibility. Within the same tumor, some cells may be highly ferroptosis-sensitive, whereas others may be protected by antioxidant programs or lipid metabolic rewiring. This intratumoral diversity may determine whether ferroptosis induction produces broad tumor control or only eliminates selected subpopulations. Ferroptosis heterogeneity also affects immune responses. If ferroptosis preferentially eliminates immune-evasive tumor cells, it may enhance immunotherapy. If resistant subclones remain and recruit suppressive myeloid cells, therapeutic benefit may be limited. Therefore, biomarker-guided stratification is necessary. Ferroptosis gene signatures, lipid peroxidation markers, GPX4/SLC7A11 expression, ACSL4 levels, CD8^+^ T-cell infiltration, and IFN-γ signatures may help identify patients most likely to benefit from ferroptosis-inducing combinations.

The therapeutic interaction between ferroptosis induction and immune checkpoint blockade is likely to vary across tumor types and cellular compartments. Although experimental studies support potential benefits in selected settings, ferroptosis does not uniformly promote antitumor immunity. The outcome may depend on tumor-cell lipid metabolism, ferroptosis resistance mechanisms, antigen-presenting cell function, and the susceptibility of effector lymphocytes to oxidative damage. Consequently, the optimal timing and sequence of ferroptosis induction and checkpoint blockade remain uncertain. Further studies should establish whether ferroptosis contributes causally to combination efficacy and identify the biological conditions under which its induction is beneficial rather than immunosuppressive.

### 5.6 The dark side: ferroptosis of immune cells and immune suppression

A key strength of the ferroptosis field is the growing recognition that ferroptosis can have opposing immune consequences depending on which cells undergo death. Ferroptosis of malignant cells may support antitumor immunity, but ferroptosis of immune cells or suppressive myeloid populations can undermine immune responses. This duality is essential for a balanced review. Ferroptosis of tumor neutrophils has been shown to cause immune suppression in cancer (22). This study is particularly important because it challenges the assumption that ferroptosis is always beneficial in oncology. Tumor-associated neutrophils and polymorphonuclear myeloid-derived suppressor cells (PMN-MDSCs) are important regulators of tumor-associated immunosuppression. If these cells undergo ferroptosis in a way that releases suppressive mediators or promotes dysfunctional inflammatory programs, the result may be impaired T-cell activity rather than tumor rejection. This finding has several implications. First, ferroptosis-inducing therapy must be evaluated at the level of cell type, not only at the bulk tumor level. Increased ferroptosis markers in a tumor sample may reflect tumor cell death, immune cell dysfunction, or suppressive myeloid remodeling (136–140). Second, ferroptosis may generate lipid mediators that shape immune responses in complex ways. Oxidized lipids can influence dendritic cell function, macrophage polarization, neutrophil activity, and T-cell survival. Third, ferroptosis in CD8^+^ T cells may contribute to immune exhaustion or loss of effector function in oxidative tumor microenvironments. These observations demonstrate that ferroptosis-associated immune outcomes cannot be interpreted without identifying the affected cellular compartment. **Figure 4** contrasts tumor-cell ferroptosis with ferroptosis-associated changes in immune-cell populations, highlighting their potentially opposing consequences for antitumor immunity.

**Figure 4:**
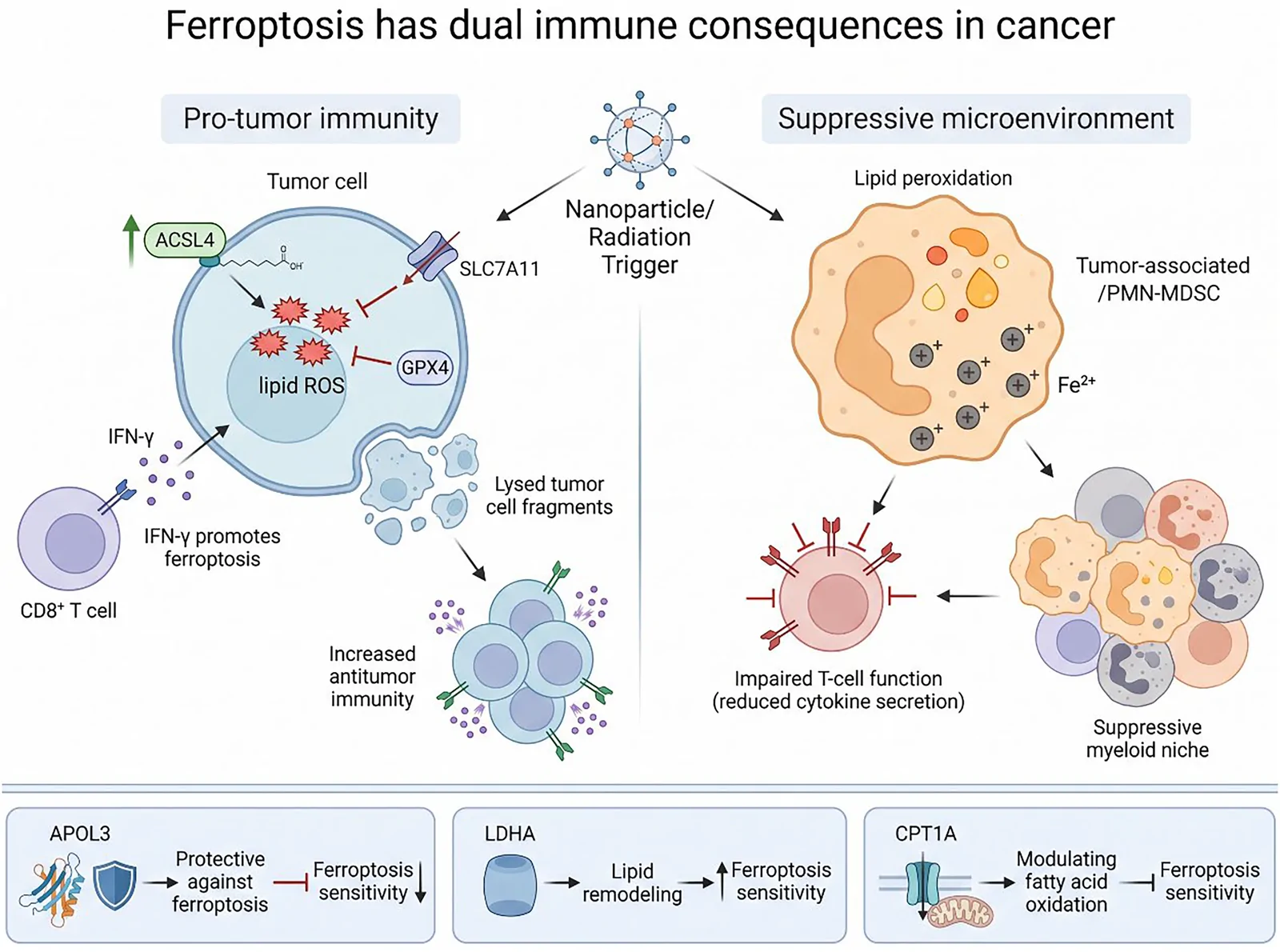
Ferroptosis exerts context-dependent effects in cancer immunity. In tumor cells, ACSL4-dependent lipid peroxidation can cooperate with CD8^+^ T-cell-derived IFN-γ to enhance tumor killing. In contrast, ferroptosis in tumor-associated neutrophils or PMN-MDSCs may reinforce immune suppression and impair T-cell function. Thus, the immune value of ferroptosis depends on the identity of the dying cell.

## 6 PANoptosis in cancer: integrated inflammatory death and immune remodeling

### 6.1 What is PANoptosis

PANoptosis is an integrated inflammatory cell death program that involves coordinated activation of pyroptosis, apoptosis, and necroptosis. Rather than representing a simple mixture of three independent death pathways, PANoptosis is mediated by interconnected signaling complexes that allow crosstalk among gasdermins, caspases, RIPK proteins, and MLKL (141–143). This concept is important because many pathological and therapeutic stimuli do not activate a single pure death pathway. Instead, tumor cells often display mixed molecular features, including caspase activation, gasdermin cleavage, RIPK3 signaling, MLKL activation, mitochondrial stress, and inflammatory mediator release.

PANoptosis is commonly associated with molecules such as ZBP1, RIPK3, RIPK1, caspase-8, caspase-1, gasdermins, and MLKL (144). Depending on the stimulus and cellular context, these components may assemble into higher-order death complexes sometimes referred to as PANoptosomes (145). These complexes coordinate multiple death-execution modules and generate broad inflammatory outputs (146). Because PANoptosis can engage several immune-stimulatory pathways simultaneously, it has attracted increasing attention in cancer immunotherapy (21).From an immunological perspective, PANoptosis may provide a stronger or more diverse danger signal than single-pathway death (147–149). Pyroptotic components may support membrane pore formation and cytokine release; necroptotic components may promote lytic DAMP release and innate immune sensing; apoptotic caspases may regulate antigen processing and gasdermin cleavage. Together, these outputs may increase tumor antigen availability, dendritic cell activation, T-cell recruitment, and checkpoint blockade responsiveness. However, PANoptosis is not automatically beneficial. Its immune consequence depends on whether it occurs in tumor cells, immune cells, stromal cells, or suppressive myeloid compartments.

### 6.2 PANoptosis and immunosuppressive niches

PANoptosis has a strong inflammatory potential, but inflammation in cancer is not always antitumor. In some contexts, PANoptosis-associated programs may contribute to immune suppression. Tumor-associated neutrophils have been reported to upregulate PANoptosis-related programs and foster an immunosuppressive microenvironment in non-small cell lung cancer (23). This observation is important because it provides a counterbalance to the idea that PANoptosis should always be therapeutically enhanced.

Tumor-associated neutrophils can promote cancer progression through multiple mechanisms, including T-cell suppression, release of proteases, production of inflammatory mediators, promotion of angiogenesis, and formation of metastatic niches. If PANoptosis-related signaling in neutrophils amplifies these suppressive circuits, the result may be immune escape rather than tumor rejection. In this context, PANoptosis may be associated with HMGB1 release, checkpoint-related pathways, or inflammatory signals that reinforce myeloid-dominated immune suppression (150–152). This finding illustrates a broader principle: the immune outcome of PANoptosis is cell-type specific. PANoptosis in malignant cells may increase antigen release and immune recognition. PANoptosis-associated programs in tumor-associated neutrophils or other myeloid cells may instead create an inflammatory but immunosuppressive niche. Therefore, future studies should distinguish tumor cell-intrinsic PANoptosis from immune cell-associated PANoptosis using single-cell sequencing, spatial transcriptomics, multiplex imaging, and lineage-specific functional models. Therapeutically, this means that PANoptosis should be programmed rather than globally induced. The goal should be to induce PANoptosis in tumor cells while preventing the emergence of suppressive myeloid inflammation. Combining PANoptosis-inducing therapies with myeloid-targeting agents, CXCR2 blockade, TIM-3/LAG3/PD-1 blockade, or dendritic cell-activating strategies may help redirect PANoptosis-associated inflammation toward effective antitumor immunity.

### 6.3 Photodynamic therapy-induced PANoptosis and checkpoint blockade

Photodynamic therapy is an attractive modality for inducing localized oxidative stress and immunogenic tumor cell death. Because it can generate ROS in a spatially controlled manner, photodynamic therapy may be particularly suitable for triggering inflammatory death within tumors while limiting systemic toxicity. Recent evidence shows that a Ce6 derivative-based photodynamic therapy can trigger PANoptosis and enhance antitumor immunity when combined with LAG3 blockade in cutaneous squamous cell carcinoma (21).

A preclinical study in cutaneous squamous cell carcinoma demonstrated that Ce6 derivative-based photodynamic therapy could engage PANoptosis-associated signaling and enhance antitumor immune responses when integrated with STING activation and LAG3 blockade (21). These findings provide proof-of-concept evidence for combining inflammatory death induction with immune checkpoint inhibition. However, the therapeutic effects were observed in experimental models, and the contribution of PANoptosis should be interpreted within the broader context of photodynamic stress, STING activation, and checkpoint inhibition. Because this approach involves multiple therapeutic components, the improvement in tumor control should not be attributed exclusively to PANoptosis. Moreover, the efficacy, safety, and optimal treatment sequence of this strategy remain unvalidated in patients. Additional studies are needed to establish whether similar benefits occur across other tumor types and whether individual components contribute independently or interactively to the observed antitumor effects.

This strategy also highlights the importance of local and temporal control. Because PANoptosis can generate strong inflammatory signals, therapies such as photodynamic treatment may allow tumor-focused induction of inflammatory death. Although localized activation may offer theoretical advantages for limiting systemic exposure, these potential safety benefits require confirmation through pharmacokinetic, biodistribution, and toxicity studies in clinically relevant models.

### 6.4 TLR4/STING-dependent PANoptosis and chemosensitization

Pattern-recognition receptor signaling is closely linked to inflammatory cell death. TLR4 and STING are two major innate immune pathways that can sense danger signals, microbial products, or cytosolic nucleic acids and induce inflammatory responses (58, 153). In cancer, activation of these pathways may enhance immunogenicity, but excessive or chronic activation may also promote inflammation-associated tumor progression (154). Therefore, therapeutic strategies that engage TLR4/STING-dependent inflammatory death require careful contextual interpretation.

Bavachinin has been reported to exert antitumor effects by activating TLR4/STING axis-dependent PANoptosis and synergistically enhancing chemosensitivity in endometrial cancer (155). This finding provides an example of how a small molecule or natural compound-derived agent can induce integrated inflammatory death and improve response to conventional therapy. By activating TLR4/STING signaling, bavachinin may promote innate immune activation, inflammatory death execution, and increased susceptibility to chemotherapy. This mechanism is conceptually important for several reasons. First, it positions PANoptosis as a bridge between innate immune sensing and therapeutic sensitization. Second, it suggests that PANoptosis induction may overcome resistance to chemotherapy by activating multiple death pathways simultaneously. Tumor cells that escape apoptosis may still be vulnerable to necroptotic or pyroptotic components of PANoptosis (155). Third, TLR4/STING activation may increase the immunogenicity of chemotherapy-induced tumor cell death, thereby supporting antigen presentation and T-cell responses. However, TLR4/STING signaling has complex roles in cancer. Acute activation may enhance antitumor immunity, whereas chronic activation may promote inflammatory tolerance, immune suppression, or tumor adaptation. Therefore, timing and dosage are likely to be critical. Therapeutic PANoptosis induction through TLR4/STING should ideally produce a short, localized, immune-stimulatory pulse rather than persistent inflammatory signaling. Combining such agents with chemotherapy, checkpoint blockade, or dendritic cell-activating strategies may improve efficacy, but biomarker-guided patient selection will be necessary.

### 6.5 Nano-immunotherapy platforms for programmable PANoptosis

Nanomedicine offers a promising strategy for controlling inflammatory cell death. Compared with free drugs, nanoplatforms can improve tumor accumulation, enable controlled release, integrate multiple therapeutic modules, and reduce systemic exposure (156). These features are particularly valuable for PANoptosis because integrated inflammatory death may require precise regulation of intensity, timing, and cellular localization.

Experimental nanomedicine platforms have been developed to coordinate inflammatory cell death induction with immune modulation. For example, a multifunctional nanozyme integrating PANoptosis-associated signaling and T-cell metabolic reprogramming improved the antitumor effects of PD-1 blockade in preclinical models (157). Another experimental strategy employed a high-entropy-engineered nanoinducer to achieve spatiotemporally controlled PANoptosis-associated responses under near-infrared stimulation (158). Although these platforms demonstrate the feasibility of integrating local cell death with immune modulation, their therapeutic effects cannot necessarily be attributed to a single death pathway because the formulations may simultaneously influence oxidative stress, innate immune signaling, and T-cell metabolism. Furthermore, their clinical translation remains limited by uncertainties concerning tumor-specific delivery, pharmacokinetics, manufacturing reproducibility, long-term safety, and therapeutic efficacy in patients (159, 160). These approaches should therefore be regarded as experimental strategies rather than established methods for overcoming resistance to immune checkpoint blockade.

Nano-immunotherapy platforms may also help solve several challenges in PANoptosis-based therapy. They can co-deliver death-inducing agents, immune adjuvants, checkpoint inhibitors, metabolic modulators, or imaging probes. They can be designed to respond to tumor acidity, ROS, glutathione, hypoxia, enzymes, or external light. They may preferentially target tumor cells or specific immune compartments, thereby reducing off-target inflammatory toxicity. Moreover, nanomedicine allows sequential or synchronized activation of multiple pathways, which is highly relevant to PANoptosis as a coordinated death network (161–163). Despite these advantages, translational barriers remain substantial. Nanoplatform complexity may complicate manufacturing, quality control, pharmacokinetic prediction, and regulatory approval. Tumor penetration is often heterogeneous, and enhanced permeability and retention effects vary across patients and tumor types. In addition, excessive PANoptosis induction may cause local tissue injury or systemic inflammation if delivery is not sufficiently tumor-selective. Therefore, future development should focus on simplified, scalable, biomarker-guided platforms with clear mechanisms of action and measurable immune endpoints.

PANoptosis provides a framework for understanding the coordinated engagement of pyroptotic, apoptotic, and necroptotic machinery under selected therapeutic conditions. However, its contribution to antitumor immunity requires careful mechanistic and cell-type-specific validation. **Figure 5** summarizes representative therapeutic approaches that engage PANoptosis-associated pathways and their potential immunological consequences.

**Figure 5:**
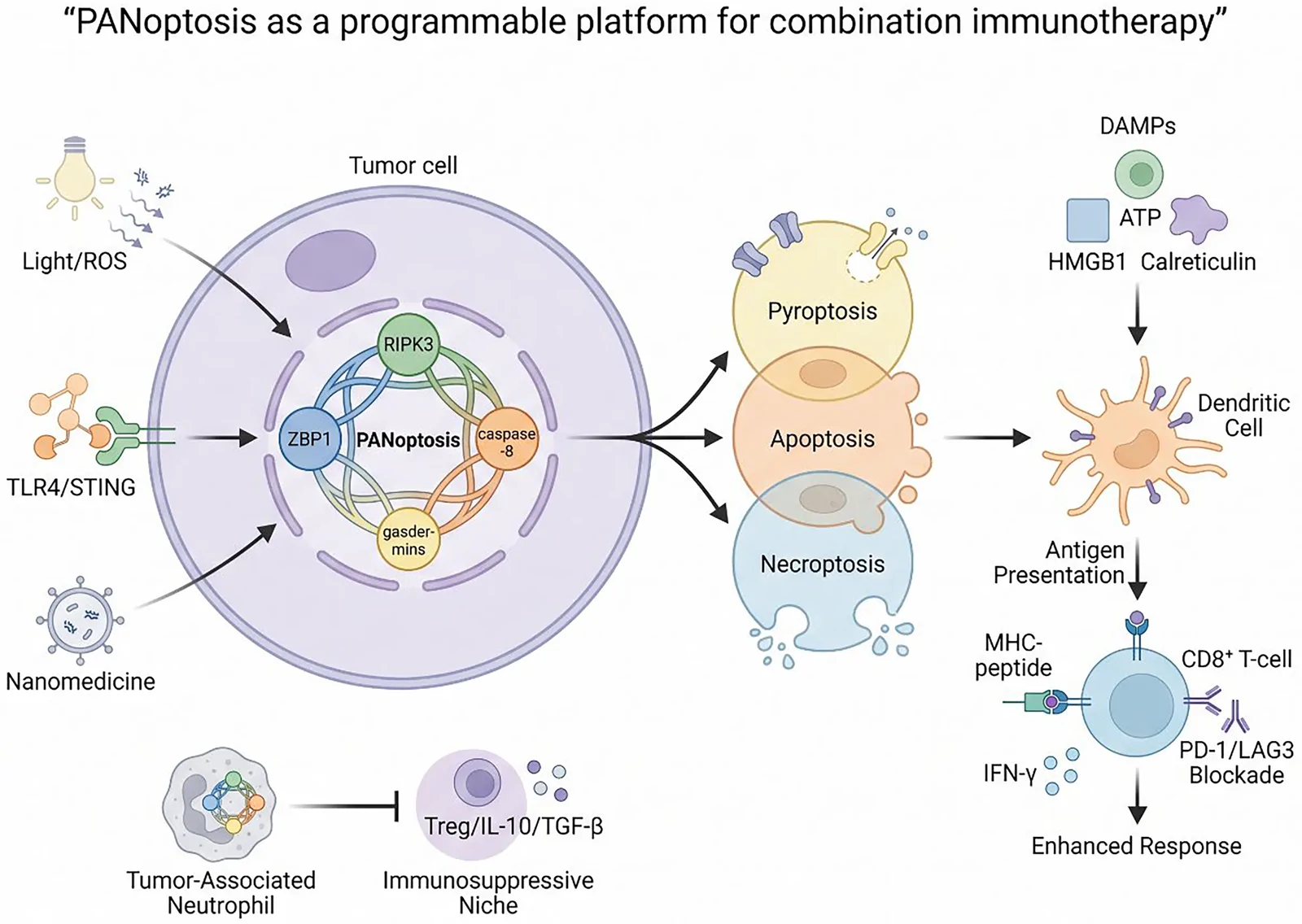
PANoptosis integrates pyroptotic, apoptotic, and necroptotic modules into a coordinated inflammatory death network. Therapeutic triggers such as photodynamic therapy, TLR4/STING activation, and nanomedicine can induce PANoptosis, leading to antigen release, dendritic cell activation, and enhanced checkpoint blockade responses. However, PANoptosis-associated programs in suppressive myeloid cells may also promote immune evasion.

## 7 Crosstalk among pyroptosis, necroptosis, ferroptosis, and PANoptosis

### 7.1 Shared upstream triggers: ROS, DNA damage, mitochondrial stress, and pattern-recognition signaling

Although pyroptosis, necroptosis, ferroptosis, and PANoptosis are usually discussed as distinct forms of regulated cell death, they are frequently activated by overlapping upstream stress signals in cancer. Therapeutic interventions such as radiotherapy, chemotherapy, photodynamic therapy, targeted metabolic inhibition, and nanomedicine-based treatments rarely induce a single isolated death pathway (18, 157). Instead, they often generate a complex stress landscape involving DNA damage, reactive oxygen species, mitochondrial dysfunction, endoplasmic reticulum stress, lipid peroxidation, and activation of innate immune sensing pathways. These shared triggers create opportunities for pathway convergence and death-mode switching.

Shared cellular stressors provide potential points of convergence among inflammatory death pathways, although exposure to the same stimulus does not necessarily indicate simultaneous activation of multiple death programs. DNA damage and mitochondrial dysfunction can generate cytosolic nucleic acid signals and engage innate immune sensing, whereas oxidative stress can influence both lipid peroxidation and caspase-associated death signaling. For example, cisplatin-induced necroptosis has been linked to mitochondrial DNA–cGAS–STING signaling (19), while PARP inhibition can enhance radiotherapy-associated ferroptosis and cGAS-dependent immune responses in colorectal cancer (125). Similarly, photodynamic therapy can engage multiple death-related signaling modules under selected experimental conditions (21). These observations suggest that molecular convergence occurs at the level of cellular stress and innate signaling, but the resulting death phenotype depends on the availability and functional activity of pathway-specific execution machinery. Distinguishing shared upstream activation from direct molecular crosstalk is therefore essential for interpreting therapy-induced inflammatory cell death.

### 7.2 Shared immune mediators do not imply equivalent immune outcomes

The presence of shared inflammatory mediators should not be interpreted as evidence that distinct cell-death programs generate equivalent antitumor immune responses (37). Although DAMP release, inflammatory cytokine production, and innate immune sensing may accompany several death modalities, their functional consequences depend on mediator abundance, cellular origin, receptor availability, and the state of responding immune cells. For example, mitochondrial DNA-associated signaling during necroptosis can support cGAS–STING-dependent immune activation under defined experimental conditions (19), whereas type I interferon signaling may contribute to protective immunity in selected necroptotic settings (46). However, neither inflammatory mediator release nor interferon induction alone establishes effective tumor-antigen cross-presentation or durable T-cell immunity.

A further distinction concerns whether inflammatory cell death occurs in malignant cells or immune compartments. Ferroptosis-associated signals may contribute to tumor-cell elimination (41), but ferroptosis involving tumor-associated neutrophils has also been linked to immunosuppression (22). Thus, apparently similar inflammatory outputs can have different biological consequences across cellular contexts. Future investigations should distinguish biochemical evidence of inflammatory death from functional demonstrations of dendritic-cell cross-presentation, tumor-specific T-cell activation, and immune-dependent tumor control (29). Such an evidence-based approach is essential for determining whether a shared inflammatory signal represents a productive immune mechanism or merely accompanies cellular damage.

### 7.3 Pathway switching and mixed death phenotypes

Pathway switching is particularly relevant in apoptosis-resistant cancers. Many tumors acquire resistance to apoptosis through p53 loss, BCL-2 family dysregulation, caspase suppression, death receptor defects, or altered mitochondrial priming (164–167). Under these conditions, alternative death pathways such as necroptosis, pyroptosis, or ferroptosis may become therapeutically valuable. For example, when caspase-8 activity is low or inhibited, RIPK1/RIPK3 signaling may shift cells toward necroptosis. When caspase-3 is activated in GSDME-expressing tumor cells, a stimulus that would otherwise induce apoptosis may be converted into pyroptosis. When antioxidant defenses are weakened, oxidative stress may drive ferroptosis instead of non-lytic death. Mixed death phenotypes can also arise because execution pathways regulate one another. Caspase-8 can suppress necroptosis by cleaving RIPK1 or RIPK3, but it can also participate in PANoptotic complexes. Caspase-3 can execute apoptosis but also cleave GSDME and promote pyroptosis. RIPK3 is central to necroptosis but may also participate in PANoptosis (168–170). ROS can promote ferroptosis while simultaneously enhancing inflammasome activation or mitochondrial damage. Therefore, inflammatory cell death is shaped by dynamic pathway competition and cooperation. This has important implications for experimental interpretation. A single marker is rarely sufficient to define the death modality. For pyroptosis, gasdermin cleavage and pore formation should be assessed together with lytic morphology and inflammatory mediator release. For necroptosis, RIPK3 and phosphorylated MLKL should be evaluated functionally. For ferroptosis, lipid peroxidation, iron dependence, GPX4/SLC7A11 status, and rescue by ferroptosis inhibitors are important. For PANoptosis, evidence of coordinated activation of pyroptotic, apoptotic, and necroptotic components is required. Without rigorous mechanistic validation, mixed death phenotypes may be oversimplified.

Importantly, the coexistence of multiple cell-death markers should be distinguished from functionally demonstrated pathway switching. Establishing a causal relationship requires pathway-specific inhibition or genetic perturbation, assessment of alternative execution mechanisms, and appropriate rescue experiments. This distinction is particularly relevant when evaluating therapies that induce overlapping stress responses or are proposed to activate integrated inflammatory death programs.

### 7.4 Why PANoptosis may represent a higher-order inflammatory death network

An important distinction must be made between PANoptosis and the nonspecific coexistence of multiple death-associated signals. PANoptosis refers to coordinated engagement of interconnected pyroptotic, apoptotic, and necroptotic machinery, rather than the simple detection of markers from several pathways in the same experimental system (144). Moreover, ferroptosis is mechanistically distinct and should not automatically be considered a component of PANoptosis (38). Therapeutic studies involving photodynamic interventions and engineered nanoplatforms illustrate the potential value of targeting integrated death programs (21, 157). Nevertheless, rigorous functional evidence is required to establish whether the observed immune effects arise from coordinated death-pathway activation or from parallel stress responses. Resolving this distinction will be essential for determining whether PANoptosis provides therapeutic advantages beyond individual death modalities.

In summary, the crosstalk among pyroptosis, necroptosis, ferroptosis, and PANoptosis reveals a central principle: inflammatory cell death is a coordinated immune-regulatory process. Shared triggers such as DNA damage, ROS, mitochondrial stress, and pattern-recognition signaling can activate multiple death modules. Shared mediators such as DAMPs, IFN-I, cytokines, and tumor antigens can shape downstream immunity. Pathway switching and mixed death phenotypes allow tumors to adapt but also provide therapeutic opportunities. PANoptosis may represent the most integrated version of this network, but its clinical value will depend on whether it can be directed toward tumor cells and synchronized with antitumor immune responses.

## 8 Cell-type specificity: who dies determines the immune consequence

### 8.1 Tumor cell death as an immune-activating event

The immunological significance of tumor-cell death depends on more than activation of a particular death pathway (29). At least three conditions should be distinguished: the ability of malignant cells to execute the death program, the capacity of local antigen-presenting cells to recognize and process death-associated material, and the preservation of functional antitumor lymphocytes (36, 39). The experimental studies discussed in Sections 3–6 illustrate how these processes can be linked under specific therapeutic conditions. However, the detection of tumor-cell death or inflammatory mediator release alone does not establish that adaptive immunity contributes to therapeutic efficacy (37). Functional assessment of antigen presentation, T-cell dependence, and immune-mediated tumor control is therefore necessary to determine whether a given death program is genuinely immune-productive.

### 8.2 Myeloid cell death as an immune-suppressive event

Inflammatory death-associated processes in myeloid compartments may have consequences that differ substantially from those occurring in malignant cells. Experimental evidence has linked ferroptosis in tumor-associated neutrophils to immunosuppression (22), while PANoptosis-associated programs in tumor-associated neutrophils have also been implicated in suppressive tumor microenvironments (23). These observations highlight an important methodological limitation: bulk measurements of death-associated markers cannot reliably identify the cellular source of inflammatory signaling. In particular, increased expression of PANoptosis-related genes should not be equated with direct evidence of PANoptotic cell death (144). Cell-type-resolved protein measurements, spatial analysis, and lineage-specific functional perturbation are needed to distinguish inflammatory death from associated transcriptional responses and determine its contribution to immune suppression.

### 8.3 T cell vulnerability to inflammatory stress

Effector T cells are central mediators of durable antitumor immunity, but they are highly vulnerable to inflammatory and metabolic stress within the tumor microenvironment. Hypoxia, nutrient deprivation, lactic acidosis, ROS accumulation, lipid peroxidation, chronic antigen stimulation, and suppressive cytokines can impair T-cell survival and function. Therefore, therapies that induce inflammatory cell death must also consider whether they preserve or damage antitumor T-cell populations. Ferroptosis is particularly relevant in this context. Although CD8^+^ T cells can induce ferroptosis in tumor cells, effector T cells themselves may be sensitive to oxidative stress and lipid peroxidation. Excessive ferroptotic stress in the tumor microenvironment may impair T-cell proliferation, cytokine production, and cytotoxic activity (171–173). Similarly, uncontrolled pyroptotic or necroptotic inflammation may increase cytokine stress, tissue damage, or suppressive myeloid recruitment, indirectly limiting T-cell function. This issue is especially important for combination therapy. A treatment may successfully increase tumor cell death but fail clinically if it simultaneously weakens T-cell immunity. Conversely, an ideal inflammatory death-inducing therapy should promote tumor antigen release, support dendritic cell activation, and preserve or enhance effector T-cell fitness. Some emerging nanomedicine strategies directly address this need by combining PANoptosis induction with T-cell metabolic reprogramming, thereby increasing tumor immunogenicity while improving T-cell function (157). Future therapeutic design should therefore move beyond measuring tumor cell death alone. Key immune endpoints should include CD8^+^ T-cell infiltration, T-cell exhaustion markers, cytokine production, proliferative capacity, metabolic fitness, memory differentiation, and resistance to oxidative stress. Protecting T cells while inducing tumor-selective inflammatory death may be essential for translating inflammatory cell death into durable immune control.

## 9 Therapeutic strategies: converting inflammatory death into durable antitumor immunity

### 9.1 Radiotherapy-based strategies

Radiotherapy is a powerful inducer of tumor stress and inflammatory cell death. Beyond DNA damage and direct cytotoxicity, radiotherapy can generate ROS, activate innate immune sensing, increase antigen release, and remodel the tumor immune microenvironment (58). These effects make radiotherapy an attractive partner for strategies designed to convert local tumor destruction into systemic antitumor immunity. The therapeutic integration of inflammatory cell death with radiotherapy requires optimization of radiation dose, fractionation, treatment sequencing, and tumor-specific death competence (174). Experimental evidence implicates GSDME-mediated pyroptosis (18), RIPK3-dependent necroptosis-associated immune signaling (19), and cGAS-linked ferroptosis in selected radiotherapy-related treatment settings (125). However, these mechanisms should not be assumed to occur simultaneously or uniformly across tumor types. Future studies should determine which radiation regimens preferentially engage specific death programs and whether their effects on antigen presentation and lymphocyte function justify combination with immunotherapy. However, radiotherapy-based inflammatory death strategies require optimization. Radiation dose and fractionation may determine whether immune activation or immune suppression predominates. Excessive radiation can damage lymphocytes and normal tissues, while insufficient stress may fail to induce immunogenic death. Future studies should define how to synchronize radiotherapy with pyroptosis, necroptosis, ferroptosis, and checkpoint blockade in a way that maximizes antigen release, dendritic cell priming, and T-cell function.

### 9.2 Chemotherapy-based strategies

Chemotherapy remains a major component of cancer treatment, and its immunological effects are increasingly appreciated. Certain chemotherapeutic agents can induce inflammatory cell death, increase DAMP release, promote antigen presentation, and sensitize tumors to immunotherapy (175, 176). Therefore, chemotherapy should not be viewed only as a cytotoxic modality but also as a potential inducer of immune-productive tumor death. Chemotherapy-associated inflammatory cell death should be evaluated according to the specific drug, tumor context, and immune consequences rather than treated as a uniform therapeutic effect. Studies of GSDME-mediated pyroptosis (40) and RIPK3-dependent necroptosis (19) demonstrate that selected chemotherapeutic interventions can engage distinct inflammatory death pathways. For translational application, a major challenge is to balance tumor-cell death induction against chemotherapy-associated damage to antigen-presenting cells and effector lymphocytes (177). Drug selection, dose intensity, treatment sequencing, and immune recovery should therefore be incorporated into the design of combination regimens (178).

### 9.3 Immune checkpoint blockade combinations: preclinical evidence and clinical limitations

Immune checkpoint blockade depends on the presence or induction of functional antitumor T-cell responses (179). Tumors that lack antigen presentation, dendritic cell priming, effector T-cell infiltration, or inflammatory signaling often respond poorly to PD-1, PD-L1, CTLA-4, or LAG3 blockade (180, 181). Inflammatory cell death provides a strategy to overcome this limitation by converting immune-cold tumors into more inflamed and immune-responsive lesions. The rationale for combining inflammatory cell death induction with immune checkpoint blockade should be based on complementary therapeutic mechanisms rather than the assumption that inflammation universally improves immunotherapy. Representative studies have reported enhanced checkpoint blockade responses following modulation of pyroptosis, necroptosis, ferroptosis, or PANoptosis-associated pathways in selected experimental models (21, 39, 41, 44). However, the optimal combination is likely to depend on whether the principal therapeutic limitation is insufficient tumor immunogenicity, defective antigen presentation, inadequate T-cell recruitment, or impaired effector function. Accordingly, combination strategies should be matched to the dominant immune resistance mechanism and evaluated using immune-functional endpoints rather than tumor-cell viability alone. Nevertheless, combination therapy must be rationally timed. Inflammatory death induction may be most useful before or during the priming phase, when dendritic cells capture antigens and initiate T-cell responses. Checkpoint blockade may then sustain the expansion and effector activity of tumor-specific T cells. If checkpoint blockade is given without adequate antigen release or innate immune activation, responses may remain limited. Conversely, if inflammatory death causes excessive myeloid suppression or T-cell damage, checkpoint blockade may fail despite increased tumor cell death (22).

An important distinction should be made between mechanistic evidence, preclinical therapeutic efficacy, and clinically validated treatment benefit. Although several studies have established associations between inflammatory death-related molecules and tumor immune responses in patient-derived specimens, these observations do not demonstrate that therapeutic induction of a specific death pathway improves clinical responses to immune checkpoint inhibitors (36). Likewise, the antitumor effects observed in immunocompetent mouse models cannot be directly extrapolated to patients because of differences in tumor heterogeneity, immune architecture, treatment exposure, and toxicity. Future clinical translation will require pharmacodynamic confirmation of the intended death pathway, identification of the affected cellular compartment, biomarker-guided patient selection, and prospective assessment of therapeutic efficacy and safety (29). Until such evidence becomes available, inflammatory cell death-based checkpoint combinations should be considered promising but predominantly investigational approaches.

### 9.4 Natural compounds and small molecules

Natural compounds and small molecules provide another route to modulate inflammatory cell death. These agents may regulate gasdermin activation, innate immune signaling, oxidative stress, mitochondrial function, or PANoptotic complexes. Their appeal lies in their potential to reshape tumor cell death programs with relatively flexible combination strategies. Small molecules and natural compound-derived agents provide pharmacological opportunities to modulate inflammatory death pathways, as illustrated by experimental studies involving curcumin (72), bavachinin (155), MDM2 inhibition (104), and CPT1A targeting (182). However, their therapeutic development requires careful separation of direct death-pathway regulation from broader effects on cellular metabolism, survival signaling, and immune function. Mechanistic specificity, achievable drug exposure, and reproducibility across tumor models should therefore be prioritized before these agents are considered for clinical combination strategies. However, natural compounds and small molecules face several challenges. Their mechanisms may be pleiotropic, dose-dependent, and context-specific. Some agents may have limited bioavailability or uncertain pharmacokinetics. Others may activate inflammatory pathways in both tumor and normal tissues. Therefore, rigorous mechanistic validation and clinically relevant dosing studies are required before these agents can be integrated into immunotherapy combinations.

### 9.5 Nanomedicine and programmable inflammatory death

Nanomedicine offers unique advantages for inflammatory cell death-based therapy. Because pyroptosis, necroptosis, ferroptosis, and PANoptosis can generate strong inflammatory signals, systemic and uncontrolled activation may cause toxicity. Nanoplatforms can improve tumor accumulation, enable controlled drug release, integrate multiple therapeutic modules, and allow external stimulus-responsive activation (156). These features make nanomedicine particularly suitable for programmable inflammatory death. The principal translational advantage of nanomedicine lies in its potential to control the cellular distribution, intensity, and timing of inflammatory death induction. Experimental platforms combining PANoptosis-associated tumor-cell death with T-cell metabolic modulation or externally triggered activation illustrate this therapeutic concept (157, 158). However, increasing platform complexity does not necessarily improve clinical feasibility. Future development should prioritize validated tumor-cell targeting, quantitative biodistribution, reproducible manufacturing, controllable pharmacodynamics, and demonstration of immune-dependent therapeutic benefit (160). Mechanisms such as ESCRT-dependent regulation of pyroptotic membrane damage, discussed in Section 3.5, may inform future delivery strategies but should be distinguished from nanomedicine approaches that have been directly experimentally validated (45). The therapeutic strategies discussed above differ in their mechanisms of death induction, spatial controllability, and potential for combination with immunotherapy. **Table 2** summarizes representative experimental approaches and their reported immune-related outcomes.

**Table 2:** Therapeutic strategies targeting inflammatory cell death to enhance antitumor immunity.

Therapeutic strategy	Primary death modality	Cancer context or model	Main mechanism	Immunological or therapeutic outcome	References
Ionizing radiation	Pyroptosis	Tumor models treated with radiotherapy	Radiation activates caspase-3–GSDME pyroptosis	Increased CD8^+^ T-cell infiltration and antitumor immunity	(18)
Neoadjuvant chemotherapy	Pyroptosis	Breast cancer	Chemotherapy induces GSDME-mediated pyroptosis	ATP/HMGB1 release, macrophage phagocytosis, T-cell activation	(40)
CDC20 inhibition	Pyroptosis	Prostate cancer	CDC20 inhibition stabilizes or facilitates GSDME-mediated pyroptosis	Enhanced antitumor immunity and anti-PD-1 response	(44)
Curcumin	Pyroptosis	Colorectal cancer	Curcumin enhances GSDME-mediated pyroptosis	Potentiates PD-1/PD-L1 checkpoint blockade	(72)
Gasdermin engineering plus membrane repair blockade	Pyroptosis	Experimental tumor models	Gasdermin-induced pore formation combined with ESCRT inhibition	Stronger pyroptotic lysis and antitumor immune activation	(45)
Cisplatin plus radiotherapy	Necroptosis	Radiation-treated tumor models	Cisplatin induces RIPK3-dependent necroptosis and mtDNA release	cGAS-STING activation, DC cross-presentation, abscopal effect	(19)
Inducible RIPK3 system	Necroptosis	Cancer cell-based immunotherapy	Engineered RIPK3-driven necroptosis	Provides safety switch and improves immunogenicity of cell-based therapy	(94)
MDM2 inhibition	Necroptosis	Immune checkpoint blockade models	MDM2 inhibition enhances RIPK3-mediated necroptosis	Synergizes with immune checkpoint blockade	(104)
PARP inhibition plus radiotherapy	Ferroptosis	Colorectal cancer	PARP inhibition enhances radiation-induced ferroptosis through cGAS signaling	Increased antitumor immune response	(125)
CPT1A targeting	Ferroptosis	Lung cancer	CPT1A inhibition disrupts lipid metabolism and induces ferroptosis	Synergizes with immunotherapy	(182)
APOL3-related metabolic regulation	Ferroptosis	Colorectal cancer	APOL3 promotes LDHA-associated ferroptosis	Enhances CD8^+^ T-cell antitumor immunity	(131)
Ferroptosis-based subtype strategy	Ferroptosis	Triple-negative breast cancer	Ferroptosis heterogeneity defines therapeutic vulnerabilities	Supports ferroptosis-inducing immunotherapy combinations	(132)
Ce6 derivative photodynamic therapy	PANoptosis	Cutaneous squamous cell carcinoma	PDT-induced ROS triggers PANoptosis	Enhances antitumor immunity with LAG3 blockade	(21)
Bavachinin	PANoptosis	Endometrial cancer	Activates TLR4/STING-dependent PANoptosis	Enhances chemosensitivity	(155)
Multifunctional nanozyme	PANoptosis	Nano-immunotherapy models	Induces PANoptosis and reprograms T-cell metabolism	Augments PD-1 inhibitor efficacy	(157)
High-entropy nanoinducer	PANoptosis	NIR-amplified cancer immunotherapy	Spatiotemporally programmed PANoptosis	Promotes DC activation, CTL response, and cancer immunotherapy	(158)

APOL3, apolipoprotein L3; CPT1A, carnitine palmitoyltransferase 1A; CTL, cytotoxic T lymphocyte; DC, dendritic cell; ESCRT, endosomal sorting complexes required for transport; GSDME, gasdermin E; LDHA, lactate dehydrogenase A; NIR, near-infrared; PARP, poly(ADP-ribose) polymerase; PDT, photodynamic therapy; ROS, reactive oxygen species.

## 10 Biomarkers and patient stratification

The clinical translation of inflammatory cell death-based therapy will depend on reliable biomarkers and rational patient stratification. Although pyroptosis, necroptosis, ferroptosis, and PANoptosis have strong mechanistic relevance, their therapeutic value is unlikely to be uniform across all tumors. Different cancers vary in death-execution machinery, innate immune sensing capacity, antigen presentation, immune-cell infiltration, stromal barriers, and metabolic state. Therefore, biomarkers are needed not only to predict whether a tumor can undergo a specific death program, but also to determine whether that death program will generate productive antitumor immunity. A useful biomarker framework should include three levels: tumor-intrinsic biomarkers, immune-context biomarkers, and treatment-response biomarkers. Tumor-intrinsic biomarkers indicate whether cancer cells possess the molecular machinery required for pyroptosis, necroptosis, ferroptosis, or PANoptosis. Immune-context biomarkers indicate whether the tumor microenvironment can convert inflammatory death into dendritic cell activation and T-cell-mediated immunity (61, 183). Treatment-response biomarkers reflect whether a given therapy actually induces immune-productive death in patients. Integrating these three levels may help identify tumors most likely to benefit from inflammatory cell death-inducing strategies.

### 10.1 Tumor-intrinsic biomarkers

Tumor-intrinsic biomarkers are the most direct indicators of whether malignant cells can execute a given inflammatory death program. For pyroptosis, key candidates include GSDME, GSDMD, caspase-3, and upstream regulators such as CDC20. GSDME is particularly important because many cancer therapies activate caspase-3, and the presence or absence of GSDME may determine whether caspase-3 activation results in apoptosis or pyroptosis. In prostate cancer, CDC20 inhibition facilitates GSDME-mediated pyroptosis and enhances anti-PD-1 therapy, suggesting that CDC20 and GSDME may together define a pyroptosis-sensitive and immunotherapy-responsive state (44). In breast cancer, chemotherapy-induced GSDME-mediated pyroptosis is associated with immune activation, supporting the potential value of GSDME as a treatment-response biomarker (40).

For necroptosis, RIPK1, RIPK3, MLKL, and MDM2 are important candidate markers. RIPK3 and MLKL represent the core execution machinery of necroptosis, whereas MDM2 may regulate the ability of tumor cells to undergo RIPK3-mediated necroptotic death. Tumors with low RIPK3 or MLKL expression may be resistant to necroptosis-inducing strategies, even when exposed to appropriate upstream stimuli. Conversely, tumors retaining functional RIPK3–MLKL signaling may be more suitable for therapies designed to induce necroptosis and enhance immune checkpoint blockade.

For ferroptosis, tumor-intrinsic biomarkers include ACSL4, GPX4, SLC7A11, APOL3, LDHA, and CPT1A. ACSL4 promotes the incorporation of polyunsaturated fatty acids into membrane phospholipids, thereby increasing susceptibility to lipid peroxidation. CD8^+^ T cells and fatty acid metabolism can induce ACSL4-dependent tumor ferroptosis, indicating that ACSL4 may reflect both ferroptosis sensitivity and immune-mediated killing potential (41). GPX4 and SLC7A11 represent major anti-ferroptotic defenses; high expression of these molecules may indicate resistance to ferroptosis. APOL3 and LDHA have been linked to ferroptosis-associated enhancement of CD8^+^ T-cell antitumor immunity, whereas CPT1A targeting can induce ferroptosis and synergize with immunotherapy in lung cancer (182). Ferroptosis heterogeneity in triple-negative breast cancer further suggests that ferroptosis markers should be interpreted in a subtype-specific manner rather than as universal predictors (132). For PANoptosis, candidate markers include ZBP1, RIPK3, caspase-8, caspase-1, gasdermins, MLKL, and HMGB1. Because PANoptosis is an integrated death program, no single marker is sufficient. Instead, a composite signature reflecting pyroptotic, apoptotic, and necroptotic modules may be more informative. In addition, HMGB1 and other DAMP-related molecules may indicate inflammatory output rather than execution machinery alone. However, PANoptosis signatures must be interpreted carefully because PANoptosis-associated programs may occur in tumor cells or in suppressive immune cells, with opposite immunological consequences.

### 10.2 Immune-context biomarkers

Tumor-intrinsic death competence does not guarantee therapeutic benefit. Even if cancer cells undergo pyroptosis, necroptosis, ferroptosis, or PANoptosis, the immune system must be capable of converting this death into antigen presentation and effector responses. Therefore, immune-context biomarkers are essential. CD8^+^ T-cell infiltration is one of the most important immune-context markers. Tumors with pre-existing CD8^+^ T-cell infiltration may be more capable of responding to inflammatory cell death-inducing therapies. In pyroptosis, increased CD8^+^ T-cell infiltration after GSDME-mediated tumor cell death suggests that gasdermin-dependent lysis may help transform the tumor immune landscape. In ferroptosis, CD8^+^ T cells are not only responders but also active inducers of tumor ferroptosis through IFN-γ and metabolic mechanisms (41). Therefore, CD8^+^ T-cell abundance, activation state, and spatial distribution should be integrated into biomarker models. Dendritic cell maturation and cross-presentation are also critical. Inflammatory cell death can release tumor antigens and DAMPs, but dendritic cells must capture these antigens and present them to T cells. Markers of dendritic cell activation, such as costimulatory molecule expression, antigen-presentation machinery, and cross-presenting DC subsets, may help predict whether inflammatory death will generate adaptive immunity. cGAS-STING activation and type I interferon signatures are closely related to this process, especially in necroptosis- and DNA damage-associated immune responses. The abundance and state of tumor-associated neutrophils and PMN-MDSCs should also be considered. Ferroptosis of tumor neutrophils can promote immune suppression rather than tumor rejection (22). Similarly, PANoptosis-associated programs in tumor-associated neutrophils can foster an immunosuppressive microenvironment in non-small cell lung cancer (23). These findings indicate that high myeloid infiltration may alter the immune consequence of inflammatory death. A tumor with strong ferroptosis or PANoptosis signals but dominant suppressive neutrophils may not respond favorably to death-inducing therapy unless myeloid suppression is also addressed.

Other immune-context biomarkers include IFN-I signatures, cGAS-STING activation, HMGB1 and ATP release, DAMP-associated gene expression, ferroptosis gene signatures, T-cell exhaustion markers, and spatial proximity between dying tumor cells and antigen-presenting cells. Importantly, these markers should ideally be evaluated using spatial and single-cell approaches. Bulk RNA or protein measurements may obscure whether death-related signals originate from tumor cells, myeloid cells, stromal cells, or lymphocytes.

### 10.3 Treatment-response biomarkers

Treatment-response biomarkers are needed to determine whether inflammatory cell death is actually induced during therapy and whether it is associated with immune activation. These biomarkers may differ according to therapeutic modality. For radiotherapy response, relevant biomarkers include GSDME expression and cleavage, caspase-3 activation, cGAS-STING signaling, IFN-I induction, CD8^+^ T-cell infiltration, and dendritic cell activation. Tumors with higher GSDME expression may be more likely to undergo radiotherapy-induced pyroptosis, whereas tumors with intact cGAS-STING signaling may be more capable of converting DNA damage into innate immune activation. Combining these markers may help predict whether radiotherapy will function as an immune-activating local treatment. For chemotherapy response, GSDME cleavage, HMGB1 release, ATP release, RIPK3/MLKL activation, and antigen-presentation markers may be useful. Chemotherapy-induced GSDME-mediated pyroptosis in breast cancer supports the possibility that gasdermin cleavage and DAMP release could serve as pharmacodynamic indicators of immune-productive chemotherapy (40). Similarly, necroptosis-associated markers may help identify chemotherapy regimens that generate immune-stimulatory tumor cell death rather than purely cytotoxic effects (19). For inflammatory cell death-based combinations with immune checkpoint blockade, candidate biomarkers should ideally capture both tumor-cell death competence and the functional state of the immune microenvironment. Molecular features such as GSDME expression, RIPK3–MLKL activity, ferroptosis-related metabolic regulators, and PANoptosis-associated signatures may provide exploratory information regarding pathway activity or experimental treatment sensitivity (23, 40). However, their ability to predict clinical responses to checkpoint blockade combinations remains unproven. For example, CPT1A-associated ferroptosis resistance has been investigated in lung cancer, but the benefit of combining CPT1A targeting with immune checkpoint inhibition was demonstrated primarily in preclinical models (182). Similarly, experimental PANoptosis-inducing nanoplatforms have shown enhanced antitumor responses when combined with PD-1 blockade, but their associated molecular signatures have not been prospectively validated as clinical predictive biomarkers (157). Future studies should evaluate these candidate markers in independent clinical cohorts and prospective trials before their use in patient selection can be recommended.

Overall, biomarker development should move beyond single-gene predictors. A more clinically useful approach would integrate tumor death machinery, immune-cell composition, innate immune activation, spatial organization, and pharmacodynamic treatment effects. Such integrated stratification may help determine which patients should receive pyroptosis-inducing radiotherapy, necroptosis-enhancing chemotherapy, ferroptosis-based immunotherapy, PANoptosis-inducing nanomedicine, or combination strategies. Because inflammatory cell death is highly context dependent, biomarker development should integrate tumor-intrinsic death machinery with immune-context and treatment-response indicators. **Table 3** summarizes candidate biomarkers that may guide patient stratification for inflammatory cell death-based cancer therapy.

**Table 3:** Candidate biomarkers for patient stratification in inflammatory cell death-based cancer therapy.

Biomarker category	Candidate biomarkers	Associated death modality	Potential clinical implication	Key caution	References
Gasdermin-dependent pyroptosis	GSDME, GSDMD, cleaved caspase-3	Pyroptosis	Predicts whether radiotherapy or chemotherapy may induce immunogenic pyroptosis	GSDME may be silenced or heterogeneous across tumors	(18, 40)
Pyroptosis regulation	CDC20, GSDME	Pyroptosis	May identify tumors sensitive to CDC20 inhibition plus anti-PD-1 therapy	Requires validation across tumor types	(44)
Natural compound response	GSDME, caspase-3, PD-1/PD-L1 status	Pyroptosis	May guide curcumin-based pyroptosis and ICB combinations	Bioavailability and dose optimization remain challenges	(72)
Necroptosis execution machinery	RIPK1, RIPK3, MLKL, phosphorylated MLKL	Necroptosis	Indicates capacity for necroptosis induction and DAMP-mediated immune activation	Low RIPK3 or MLKL expression may limit efficacy	(19, 46)
Necroptosis-enhancing targets	MDM2, RIPK3, MLKL	Necroptosis	May guide MDM2 inhibition combined with checkpoint blockade	Necroptosis must be linked to immune activation, not only tumor killing	(104)
Ferroptosis sensitivity	ACSL4, GPX4, SLC7A11, lipid ROS	Ferroptosis	Predicts susceptibility to CD8^+^ T-cell-induced or drug-induced ferroptosis	Bulk ferroptosis markers may not reveal which cell type is dying	(41)
Ferroptosis metabolic regulation	APOL3, LDHA, CPT1A	Ferroptosis	May identify tumors suitable for metabolic ferroptosis induction and immunotherapy	Metabolic targets may also affect immune-cell fitness	(131, 182)
Ferroptosis subtype	Ferroptosis gene signatures, GPX4/SLC7A11/ACSL4 pattern	Ferroptosis	Supports subtype-specific immunotherapy combinations, especially in TNBC	Intratumoral heterogeneity may limit single-marker prediction	(132)
Myeloid ferroptosis risk	TANs, PMN-MDSCs, neutrophil ferroptosis markers	Ferroptosis	Helps identify cases where ferroptosis may promote immune suppression	Ferroptosis induction may be harmful if dominant in suppressive myeloid cells	(22)
PANoptosis signature	ZBP1, RIPK3, caspase-8, caspase-1, gasdermins, MLKL, HMGB1	PANoptosis	May predict response to PANoptosis-inducing therapies	Composite signatures are needed; single markers are insufficient	(21, 155)
PANoptosis-associated immune suppression	TAN abundance, HMGB1-related signaling, checkpoint-associated programs	PANoptosis	May identify tumors requiring myeloid reprogramming before PANoptosis induction	PANoptosis in TANs may reinforce immune escape	(23)
Nano-immunotherapy response	PANoptosis signature, STING activation, T-cell metabolic state, PD-1 axis	PANoptosis	May guide nanomedicine plus PD-1 blockade strategies	Requires pharmacodynamic and spatial validation	(157, 158)

ACSL4, acyl-CoA synthetase long-chain family member 4; GSDMD, gasdermin D; GSDME, gasdermin E; GPX4, glutathione peroxidase 4; ICB, immune checkpoint blockade; MLKL, mixed lineage kinase domain-like protein; PMN-MDSCs, polymorphonuclear myeloid-derived suppressor cells; RIPK3, receptor-interacting protein kinase 3; TANs, tumor-associated neutrophils; TNBC, triple-negative breast cancer.

## 11 Challenges and future perspectives

Despite rapid progress, the therapeutic use of inflammatory cell death in cancer remains at an early stage. Most evidence comes from experimental models, including tumor cell lines, syngeneic mouse models, xenografts, genetically engineered systems, and nanomedicine platforms. Although these studies provide important mechanistic insight, several challenges must be addressed before inflammatory cell death can be reliably translated into clinical oncology.

### 11.1 Death intensity versus immune quality

A major conceptual challenge is distinguishing death intensity from immune quality. Strong tumor cell death does not necessarily generate effective antitumor immunity. Massive uncontrolled cell death may produce necrotic debris, chronic inflammation, tissue injury, or suppressive myeloid recruitment. In contrast, a more controlled form of inflammatory death may generate sufficient antigen release and danger signaling while preserving immune function. Therefore, the therapeutic goal should not be to maximize cell death at any cost. Instead, the goal should be to induce immune-productive death. Immune-productive death can be defined as a death program that releases tumor antigens and DAMPs, activates dendritic cells, promotes T-cell priming, preserves effector lymphocyte function, and avoids dominant immunosuppressive inflammation. This distinction is especially important for ferroptosis and PANoptosis. Ferroptosis in tumor cells may enhance immunity, but ferroptosis in tumor-associated neutrophils may promote immune suppression (22). PANoptosis in malignant cells may increase immunogenicity, whereas PANoptosis-associated programs in neutrophils may reinforce an immunosuppressive niche (23). Future studies should therefore measure not only cell viability and tumor size but also immune quality. Relevant endpoints include DAMP release, antigen presentation, dendritic cell maturation, CD8^+^ T-cell infiltration, T-cell cytotoxicity, T-cell memory, myeloid suppression, cytokine balance, and systemic immune protection. Without these immune endpoints, it is difficult to distinguish effective immunogenic cell death from inflammatory damage.

### 11.2 Spatial and temporal control

Inflammatory cell death is most likely to be beneficial when it occurs in the right place and at the right time. Ideally, death should be induced selectively in tumor cells within the tumor microenvironment, where released antigens and danger signals can be captured by antigen-presenting cells. Systemic induction of inflammatory death may damage normal tissues, activate unwanted inflammation, or impair immune cells.

Spatial control can be achieved through local therapies and targeted delivery. Radiotherapy can focus stress within tumor lesions. Photodynamic therapy can generate ROS in illuminated tumor regions and trigger PANoptosis in a localized manner (21). Nanomedicine platforms can improve tumor accumulation and enable controlled release of death-inducing agents. Engineering pyroptosis by preventing ESCRT-dependent membrane repair illustrates how delivery systems may intensify inflammatory tumor cell death while limiting systemic exposure (45). Multifunctional nanozymes and high-entropy-engineered nanoinducers further show how nanoplatforms can induce PANoptosis and enhance immunotherapy through programmed or externally amplified activation (157, 158). Temporal control is equally important. Inflammatory death may be most useful during the immune-priming phase, when antigen release and dendritic cell activation are needed. Checkpoint blockade may be more effective after or during this priming phase, when tumor-specific T cells are expanding and becoming functionally engaged. If inflammatory death occurs too early without immune support, antigens may be cleared without effective priming. If it occurs too late or too broadly, it may promote tissue damage or suppressive inflammation.

Future therapeutic designs should therefore focus on spatiotemporally controlled death programs. External stimuli such as light, ultrasound, radiation, magnetic fields, or near-infrared activation may be combined with tumor-targeted nanomedicine to induce inflammatory death precisely. This approach may improve the therapeutic index and reduce the risk of systemic inflammatory toxicity.

### 11.3 Cell-type-specific targeting

Cell-type specificity is one of the most important challenges in this field. Bulk tumor analysis often cannot identify which cells are undergoing inflammatory death. This is a major limitation because the same death pathway may have opposite effects depending on the dying cell type. Tumor cell death may promote antigen release and immune activation, whereas myeloid cell death may promote immune suppression. T-cell death may directly undermine therapeutic efficacy.

Ferroptosis illustrates this problem clearly. Tumor cell ferroptosis may enhance immune-mediated tumor control, but ferroptosis of tumor-associated neutrophils can cause immune suppression (22). PANoptosis provides another example: PANoptosis-associated signaling in tumor-associated neutrophils may foster an immunosuppressive microenvironment in non-small cell lung cancer (23). These studies demonstrate that measuring a death pathway at the whole-tumor level may be insufficient and potentially misleading. Future studies should use single-cell and spatial technologies to localize inflammatory death. Single-cell RNA sequencing can identify death-related transcriptional programs in malignant, myeloid, stromal, and lymphoid compartments. Spatial transcriptomics and multiplex imaging can reveal whether dying tumor cells are located near dendritic cells, T cells, blood vessels, hypoxic regions, or suppressive myeloid niches. Functional validation using lineage-specific genetic models will be necessary to determine causality. Therapeutically, cell-type-specific targeting may require tumor-selective promoters, antibody-drug conjugates, ligand-modified nanoparticles, local activation systems, or microenvironment-responsive delivery. It may also require combination with myeloid reprogramming strategies to prevent suppressive inflammation. The future of inflammatory cell death therapy will depend on inducing the right kind of death in the right cell population.

### 11.4 Combination timing with immunotherapy

The timing and sequence of inflammatory cell death-inducing therapies relative to immune checkpoint blockade represent important but incompletely understood determinants of treatment response. Although preclinical observations suggest that treatment-induced inflammatory signaling may influence subsequent immune activation, the optimal sequencing of death-inducing interventions and checkpoint inhibitors has not been established clinically. Moreover, the most appropriate schedule may vary according to tumor type, treatment modality, immune status, and the kinetics of cell death and immune recovery.

As a conceptual framework for future investigation rather than a clinically validated treatment schedule, combination therapy may be considered in three overlapping phases: immune priming, effector expansion, and immune memory formation. The first is the priming phase. During this phase, radiotherapy, chemotherapy, photodynamic therapy, ferroptosis induction, or PANoptosis-inducing nanomedicine can be used to release tumor antigens, DAMPs, nucleic acid ligands, and inflammatory mediators. The goal is to activate dendritic cells and initiate tumor-specific T-cell priming. The second is the expansion phase. During this phase, immune checkpoint blockade, such as PD-1/PD-L1 or LAG3 inhibition, may enhance expansion and effector function of newly primed T cells. This is supported by studies showing that pyroptosis induction, necroptosis enhancement, ferroptosis targeting, and PANoptosis induction can improve responses to checkpoint blockade (21, 44, 157, 182). In this phase, maintaining T-cell metabolic fitness and preventing exhaustion are essential. The third is the memory phase. Durable tumor control requires long-term immune surveillance. After initial tumor regression, therapies should ideally support memory T-cell formation, tissue-resident immune surveillance, and antigen spreading. Excessive or prolonged inflammatory death during this phase may be harmful if it causes chronic inflammation or T-cell dysfunction. Therefore, inflammatory death induction may need to be pulsed, localized, or discontinued once sufficient immune priming has been achieved.

Prospective studies should test whether these proposed phases can inform treatment scheduling and whether sequential, concurrent, or adaptive combinations provide superior efficacy and safety in defined patient populations. Instead of simply administering death-inducing agents and checkpoint inhibitors concurrently, future studies should compare sequential, staggered, and adaptive schedules. Biomarkers such as DAMP release, IFN-I signatures, dendritic cell activation, T-cell expansion, and exhaustion markers may help guide timing.

### 11.5 Translational caution

Although the field is promising, several translational barriers remain. Many current studies rely on mouse tumor models, which may not fully reproduce the immune architecture, stromal complexity, antigen heterogeneity, and treatment history of human cancers. Cell-line models often lack the spatial and cellular diversity of patient tumors. Nanomedicine platforms may show strong preclinical efficacy but face challenges in manufacturing, pharmacokinetics, biodistribution, tumor penetration, safety, and regulatory approval.

Dose optimization is another major issue. The dose required to induce inflammatory cell death in tumor cells may also damage normal tissues or immune cells. Systemic induction of pyroptosis, necroptosis, ferroptosis, or PANoptosis could produce toxicity if not properly targeted (126, 184, 185). Therefore, therapeutic windows must be carefully defined. Pharmacodynamic biomarkers will be needed to confirm pathway activation in tumors without excessive systemic inflammation. Patient selection is equally important. Not all tumors express GSDME, RIPK3, MLKL, ACSL4, or other required death-execution molecules. Some tumors have defective cGAS-STING signaling, impaired antigen presentation, or severely exhausted T-cell compartments. In such tumors, inducing inflammatory death may not translate into effective immunity unless these additional barriers are addressed. Therefore, clinical development should incorporate biomarker-guided enrollment and immune monitoring.

Finally, inflammatory cell death should be evaluated as part of a tumor ecosystem rather than as a single molecular event. The same pathway may be beneficial in one tumor context and harmful in another. Successful translation will require integration of tumor biology, immune contexture, spatial organization, treatment timing, and patient-specific biomarkers.

## 12 Conclusion

Inflammatory cell death is reshaping the way cancer therapy is understood. Pyroptosis, necroptosis, ferroptosis, and PANoptosis are not merely alternative mechanisms of tumor cell elimination. They are immune-regulatory processes that connect therapeutic stress, innate immune sensing, antigen release, dendritic cell activation, and T-cell-mediated antitumor responses. Through DAMPs, cytokines, type I interferons, lipid peroxidation signals, and cGAS-STING activation, dying tumor cells can communicate with the immune system and influence whether therapy produces local cytotoxicity or systemic immune control.

The key message emerging from recent studies is that the mode and context of death matter. GSDME-mediated pyroptosis can convert radiotherapy or chemotherapy into an immune-stimulatory event. RIPK3–MLKL-mediated necroptosis can engage mitochondrial DNA sensing, cGAS-STING activation, type I interferon responses, and dendritic cell cross-presentation. Ferroptosis links tumor lipid metabolism with CD8^+^ T-cell-mediated killing, but it can also promote immune suppression when it occurs in tumor-associated neutrophils. PANoptosis integrates multiple inflammatory death modules and offers opportunities for photodynamic therapy, innate immune activation, nanomedicine, and checkpoint blockade, but its outcome remains highly cell-type dependent. Therefore, the future of this field should move from simply inducing death to designing immune consequences. Effective therapeutic strategies will need to induce inflammatory death selectively in tumor cells, preserve dendritic cell and T-cell function, avoid suppressive myeloid remodeling, and synchronize death induction with immune checkpoint blockade or other immunotherapies. Biomarkers will be essential for identifying tumors with intact death-execution machinery, functional innate immune sensing, and a permissive immune microenvironment.

Inflammatory cell death provides a valuable conceptual framework for understanding the interaction between tumor-cell elimination and antitumor immunity. Although preclinical studies have identified potential opportunities for combining pyroptosis, necroptosis, ferroptosis, or PANoptosis-associated interventions with immune checkpoint blockade, the clinical efficacy of these strategies remains to be established. Future investigations should prioritize tumor-specific mechanistic validation, cell-type-resolved immune assessment, biomarker development, and prospective evaluation of combination efficacy and safety. Translating inflammatory cell death into clinically meaningful immunotherapy will require a shift from demonstrating enhanced tumor killing in experimental models toward establishing reproducible, immune-dependent therapeutic benefits in patients.
